# Transcriptome analysis of lentil (*Lens culinaris* Medikus) in response to seedling drought stress

**DOI:** 10.1186/s12864-017-3596-7

**Published:** 2017-02-27

**Authors:** Dharmendra Singh, Chandan Kumar Singh, Jyoti Taunk, Ram Sewak Singh Tomar, Ashish Kumar Chaturvedi, Kishor Gaikwad, Madan Pal

**Affiliations:** 1 Division of Genetics, Indian Agricultural Research Institute, New Delhi, 110012 India; 2 Division of Plant Physiology, Indian Agricultural Research Institute, New Delhi, 110012 India; 3 National Research Centre on Plant Biotechnology, ICAR, New Delhi, 110012 India

**Keywords:** Differential gene expression, Drought stress, Lentil, Transcriptomics

## Abstract

**Background:**

Drought stress is one of the most harmful abiotic stresses in crop plants. As a moderately drought tolerant crop, lentil is a major crop in rainfed areas and a suitable candidate for drought stress tolerance research work. Screening for drought tolerance stress under hydroponic conditions at seedling stage with air exposure is an efficient technique to select genotypes with contrasting traits. Transcriptome analysis provides valuable resources, especially for lentil, as here the information on complete genome sequence is not available. Hence, the present studies were carried out.

**Results:**

This study was undertaken to understand the biochemical mechanisms and transcriptome changes involved in imparting adaptation to drought stress at seedling stage in drought-tolerant (PDL-2) and drought-sensitive (JL-3) cultivars. Among different physiological and biochemical parameters, a significant increase was recorded in proline, glycine betaine contents and activities of SOD, APX and GPX in PDL-2 compared to JL-3while chlorophyll, RWC and catalase activity decreased significantly in JL-3. Transcriptome changes between the PDL-2 and JL-3 under drought stress were evaluated using Illumina HiSeq 2500 platform. Total number of bases ranged from 5.1 to 6.7 Gb. Sequence analysis of control and drought treated cDNA libraries of PDL-2 and JL-3 produced 74032, 75500, 78328 and 81523 contigs, respectively with respective N50 value of 2011, 2008, 2000 and 1991. Differential gene expression of drought treated genotypes along with their controls revealed a total of 11,435 upregulated and 6,934 downregulated transcripts. For functional classification of DEGs, KEGG pathway annotation analysis extracted a total of 413 GO annotation terms where 176 were within molecular process, 128 in cellular and 109 in biological process groups.

**Conclusion:**

The transcriptional profiles provide a foundation for deciphering the underlying mechanism for drought tolerance in lentil. Transcriptional regulation, signal transduction and secondary metabolism in two genotypes revealed significant differences at seedling stage under severe drought. Our finding suggests role of candidate genes for improving drought tolerance in lentil.

**Electronic supplementary material:**

The online version of this article (doi:10.1186/s12864-017-3596-7) contains supplementary material, which is available to authorized users.

## Background

Lentil (*Lens culinaris* Medikus), a self pollinating crop with an approximate genome size of 4 Gbp [1] is an important legume which provides quality protein, carbohydrates, fibre and minerals for the humans and fodder for livestock. It is a moderately drought tolerant crop [2], but the yield is drastically reduced with increased drought stress. As water availability is important for crop growth and productivity, drought stress at critical stage with high severity can impose a threat to world food security. It ranks as the single most common cause of severe food shortages mostly in the developing parts of the world and represents far-reaching natural trigger of malnutrition and famine [3]. The mechanisms for drought tolerance in plants are very complex and highly variable [4]. Although, the tolerance to drought stress in lentil varies considerably among genotypes yet genomic information pertaining to drought stress is limited in this crop. For germplasm enhancement and to develop hardy lentil plants with tolerance to drought stress, understanding of molecular mechanisms governing response towards drought stress is necessary.

Drought stress induces a number of biochemical and physiological responses which are controlled by a number of genes at molecular, cellular and whole plant level that helps in maintaining water and ionichomeostasis and protect the plant from wilting and destined death. This can be achieved by maintaining osmotic compatibility within the cell, reconstruction of primary and secondary metabolism and by restoration of proteins in their native folded tertiary structure. Most well reported mechanisms of drought stress tolerance has been related to accumulation of n metabolites like proline, glycine betaine, soluble carbohydrates antioxidants etc. which help in maintaining the vital properties of the cell [5–8]. Several major classes of genes have been documented whose expressions are altered under drought stress. Most prominent among them are those involved in cellular metabolism, including cellular detoxification e.g., aldehyde dehydrogenase family genes; genes involved in cellular transport and signal transduction e.g. for ABA responses; genes encoding transcription factors which are involved in transcriptional regulation and genes for hydrophilic and heat-soluble proteins e.g., *late embryogenesis abundant* (*lea*) genes, etc. [9]. Functional genes comprised of heat shock proteins, facilitating protein refolding and stabilizing polypeptides and membranes under drought stress. Endogenous abscisic acid (ABA), content increases under drought stress which protects the plant from immediate desiccation by stomatal closure [10, 11]. ABA has been shown to regulate expression of few genes under drought stress [12].

Recent developments of Next Generation Sequencing (NGS) technologies have enabled mass sequencing of genomes and transcriptomes, which produce a vast array of genomic information [13]. Genome wide expression studies provide to breeders a framework of dataset to understand the molecular basis of complex traits. Using NGS technology, Bett et al. have used lentil cultivar, CDC Redberry to develop an initial draft of 23x coverage which covered over half the lentil genome (2.7 Gb of the expected 4.3 Gb) [14]. Single nucleotide polymorphisms (SNPs) derived from this draft are available for use in molecular breeding of lentil. Kaur et al. have also performed sequence analysis in lentil using second generation sequencing technology and have developed a collection of expressed sequence tags (ESTs) [15]. In other legumes like chickpea, transcriptome analysis under drought stress has already been undertaken. Hiremath et al. used Roche/454 and Illumina Solexa to identify drought responsive genes and gene based molecular markers including simple sequence repeats (SSRs), SNPs and conserved ortholog set (COS) in chickpea [16]. A number of transcription factor families and defence related genes were identified in peanut under drought stress, using transcriptome analysis [17]. Wu et al. have found differentially expressed genes (DEGs) between terminal drought and optimal irrigation treatments in two different genotypes of common bean *i.e.* Long 22-0579 and Naihua which were functionally associated with drought stress [18]. However, information regarding transcriptomic changes under drought stress in lentil is very limited; therefore, to deduce pathways involved in drought stress response, expression study in contrasting genotypes of lentil is essential. Significant changes in gene expression are difficult to appraise without comparison. Therefore, PDL-2 and JL-3 which are two contrasting genotypes for drought tolerance, PDL-2 being drought tolerant and JL-3, drought sensitive were used in this study [19]. This study was undertaken to understand the biochemical mechanisms associated with adaptation to drought stress at seedling stage and to identify differentially expressed genes in contrasting lentil genotypes under drought stress using IlluminaHiSeq2500 platform.

## Methods

### Plant material, cultivation and drought stress treatment

Two lentil genotypes: drought tolerant (PDL-2) and drought sensitive (JL-3) were included in this study. These two genotypes were selected on the basis of previous studies, reporting PDL-2 as a drought tolerant breeding line and JL-3 was drought sensitive on the basis of seedling survivability [19]. PDL-2, a breeding line derived from a cross between ILL-590 and ILL-7663, was obtained from International Center for Agricultural Research in the Dry Areas (ICARDA), Syria. JL-3 is a released variety of Central Zone of India and it is selected from landrace of Sagar district of Madhya Pradesh, India.

### Hydroponic experiment

The hydroponic experiment was conducted at National Phytotron Facility, Indian Agricultural Research Institute (IARI), New Delhi, India in a completely randomized block design with three replications. Air temperature in the controlled environment was 22/18 °C (2 °C) day/night; photoperiod was 10/14 h light/dark; and the relative humidity was approximately 45%.

Drought tolerance was evaluated by the protocol of Singh et al. in a nutrient solution culture [20]. Seeds were disinfected with 1% sodium hypochlorite for 2–3 min and rinsed thoroughly with distilled water and then germinated on filter paper. One week old seedlings were transferred to hydroponic medium (KNO_3_ (0.5 mM), Ca (NO_3_)_2_.4H_2_0 (0.5 mM), MgSO_4_.7H_2_O (0.2 mM), KH_2_PO_4_ (0.1 mM), KCl (50 μM), H_3_BO_3_ (46 μM), Fe-EDTA (20 μM), MnCl_2_.4H_2_O (2 μM), ZnSO_4_.7H_2_O (1 μM), CuSO_4_.5H_2_O (0.3 μM) and NaMoO_4_.2H_2_O (0.5 μM) [21]. Drought stress was given one week after transplantation of seedlings. Two environments were created: Drought stress- the seedlings were exposed to air for 4 h for a period of 3 d. Control- the plants were kept in the nutrient solution for the entire period (3 d) of development without interruption. The pH of nutrient solution was adjusted at 6.5 with 1 M HCl or 1 M KOH. The solution was regularly aerated by an aquarium air pump and was replaced on alternate days. After 3 d of treatment, both the genotypes were rated for drought tolerance based on scale suggested by Singh et al. [20]. Data on relative water content (RWC), membrane stability index (MSI), photosynthetic pigments (total chlorophyll), proline, glycine betaine (GB), lipid peroxidation and antioxidant activities were taken after 3 d of drought stress treatment as described previously by Singh et al. [19, 22, 23]. The experiments were conducted in a completely randomized block design with three replications comprising twelve seedlings per replication.

### Total ribonucleic acid (RNA) extraction and purification

Leaf samples were taken from the 12 seedlings for each genotype under control and drought stress treatments. Total RNA was extracted from leaves of drought tolerant line, PDL-2 and drought sensitive variety, JL-3 from both control and treated samples using QIAGEN RNeasy Plant Mini Kit. Ribosomal RNA was removed from total RNA by using Epicentre RiboZerorRNA removal Kit and Agencourt RNA clean XP Kit. Equal amounts of total RNA extracted from each seedling of each genotype were pooled together.

### Complementary DNA (cDNA) library construction and sequencing

Strand specific cDNA library was constructed using TruSeq RNA library preparation kit following the manufacturer’s instructions. The quality of cDNA libraries was tested using Agilent 2100 bioanalyzer and quantified cDNA was subjected to purification using AMPure XP beads. Purified cDNA was then end repaired, using 3′ to 5′ exonuclease activity of End Repair Mix which removed 3′ overhangs and filled 5′ overhangs through its polymerase activity. cDNA was then polyadenylated and multiple indexing adapters were ligated to its end. For enrichment of cDNA in the library, polymerase chain reaction (PCR) was performed which selectively amplified those fragments that have adapter molecules on both the ends. The established cDNA libraries were sequenced using Illumina HiSeq2500 platform (Illumina Inc., San Diego. CA, USA) to generate 2 × 100 base paired end-reads.

### Sequence analysis

Raw Fastq files obtained from the sequencer were checked for quality parameters of the sequences *viz*. base quality score distribution, average base content per read and GC distribution in the reads. The base quality score distribution and average base content per read were inferred using Phred quality score. The raw Fastq files were trimmed before performing de novo transcriptome assembly. First two bases and last ten bases were removed from all the reads. Fastq-mcf tool was used for removing adapter sequences. Reads of average quality score of less than 20 were also filtered out. Trinity with default options were used to assemble trimmed reads. Transcripts > = 200 bp were focussed for transcription expression estimation and downstream annotation. The trimmed reads were aligned to the assembled transcriptome (length > =200 bp) using Bowtie programme.

### Screening and annotation of DEGs

Differential gene expression studies were performed using DESeq program. Transcripts having read counts > =1 and adjusted *p* value < = 0.05 were chosen for differential gene expression analysis. The read counts, transcript expression in Fragments Per Kilobase of transcript per Million mapped reads (FPKM) were evaluated in each stage. Edward plots were plotted to elucidate comparison of contigs in all the samples, both for upregulated and downregulated contigs. DEGs between treatments were also identified based on the FPKM value using with the edgeR program. We combined the statistical test with the multiple-hypothesis-testing correction method Benjamini and Hochberg [24], which calculates the False Discovery Rate (FDR), to qualify statistically significant, differentially expressed genes by avoiding inflation of type-1 errors.

The assembled transcript was annotated using CANoPI (Contig Annotator Pipeline). Assembled transcripts were compared with National Center for Biotechnology Information (NCBI) non-redundant protein database using BLASTx (Basic Local Alignment Search Toolx) programme. Matches with E-value < = 10^-5^ and similarity score > = 40% were used for further annotation. For organism annotation, the top BLASTX hit of each transcript was studied and organism name was extracted. Gene and protein annotation was done as perthe matched transcripts. Among the total significant BLASTX hit transcripts 94,694 were annotated using UniProt (Universal Protein Resource) database and for the remaining ones, NCBI predicted protein annotation was done. Transcripts with proper gene name from UniProt and differentially expressed transcripts were shown with heat map. Gene Ontology (GO) terms for transcripts were extracted and were categorized into molecular function, biological process and cellular component categories. Enrichment analysis of Kyoto Encyclopedia of Genes and Genomes (KEGG) metabolic pathways was performed using KOBAS (KEGG Orthology Based Annotation System). Reactome analysis was performed to predict the pathway information for DEGs.

### Differential expression verification by Quantitative real-time PCR (qRT-PCR)

Accuracy of transcriptome sequencing data was validated by qRT-PCR. RNA was extracted from leaves of four biological replicates using QiagenRNeasy Plant Mini Kit. Quantification of RNA was done using Nano drop Spectrophotometer. Total RNA sample was reverse transcribed using Biorad cDNA synthesis Kit. Variable amount of total RNA for different samples was taken for final reaction volume of 20 μl. Normalization of all the cDNA samples was done so as to equalize concentration of 90 ng/μl. The primer sequences were designed using Primer3Plus and are listed in Table 1. The β-tubulin gene was used as a reference gene to normalize all the data. The 25 μl PCR reaction mixture was comprised of 4 μl diluted cDNA, 4 μl each of forward and reverse primers, 12 μl Evagreen dye. PCR amplification was carried out at 50^o^ C for 2 min, 95^o^ C for 10 min and 40 cycles consisting of 95^o^ C for 15 s and 60^o^ C for 1 min as run parameters. The relative quantification method 2^-(ΔΔCT)^ was used to calculate and calibrate the expression level of target genes in different treatments.

**Table 1:** Primers used in RT PCR validation

Gene	Primer	Forward sequence	Reverse sequence
ALDH2B4 ALDH2 At3g48000 T17F15.130	DR_LC_01	TTCAACCAGGGGCAATGTTG	ATGCGCGTGCCTTTGATTTC
At2g42790	DR_LC_02	GCGCTTTCCATCTTTCATCCTG	AGCTGCTGCAATTGTTGGTG
MT2A At3g09390 F3L24.28	DR_LC_03	ACTTTTGTCTTGGGCGTTGC	ATCCACACTTGCAAGCATCG
SRG1 At1g17020 F20D23.28 F6I1.30	DR_LC_04	AACCCCTCCCAAATGCTTTC	AATTCACAACTCCGCGATGC
HSP17.6B At2g29500 F16P2.12	DR_LC_07	TTACGAGGAGGTTTAGGTTGCC	AACACACCATTCTCCATCGC
LEA4-5 At5g06760	DR_LC_12	ACACATCAGATGTCGGCTCTG	TCCAGTGTTCCTTCCAATCGG
SDH1-1 At5g66760 MSN2.16	DR_LC_17	AAGAATGGTGGCCGGAAAAC	TGAAACCAACCGCAAAACCG
SHM1 SHMT1 STM At4g37930 F20D10.50	DR_LC_18	TGGCCAAAACAGCTTAACGC	TTGCATCACCGACACAGATG
AFP3 At3g29575 MWE13.5	DR_LC_10	TATGTTGCAGGCTCATGTCG	AGCCTTGTTTTCGCAAGTGG
At2g38470	DR_LC_19	TACAAGTGCACAACCATCGG	TGCTCTGTTTGTGGCGTAAC

### Filtering and alignment of SSRsand SNPs

SNPs were predicted using Samtools mpileup and custom scripts which call the variants based on read depth. A minimum read depth of 10 was used to filter heterozygous loci and false positive SNPs. In parallel to this, GATK toolkit was also considered to call variants using haplotype caller command version 3.6-0 for SNP calling keeping default parameters. Misa software was used for filtering SSRs from high quality filtered reads from both the genotypes that were aligned to the contigs and primers were designed using Primer3 software. Minimum primer size of the SSR primers designed using primer3 software was 15 and maximum primer size was 21 with optimal primer size of 18. Estimated product sizes from these SSRs were in the range of 100 to 300 bp.

## Results

### Variation in wilting and seedling survival

**Fig. 1:**
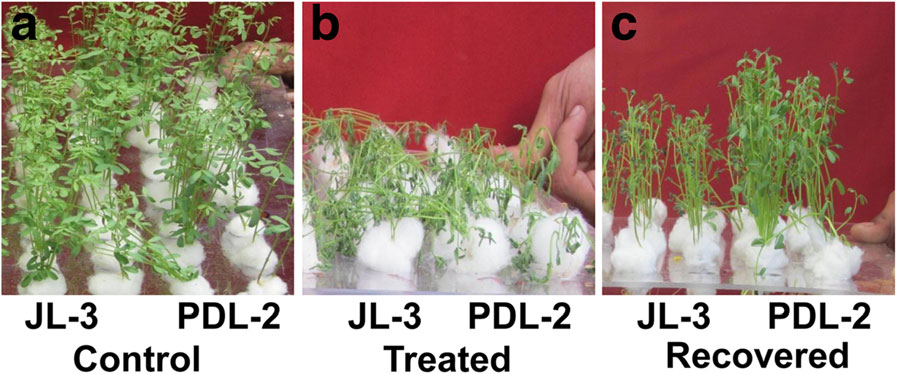
Phenotypic responses of genotypes, JL-3 (Sensitive) and PDL-2 (Tolerant)for drought stress. Control (**a**), Air exposure for 4 h and 2 days (**b**), Recovery after 12 h in nutrient solution (**c**)

Differences between genotypes under control and drought stress were found significant for wilting and seedling survival (Fig. 1a and c). The effects of stress were first observed on the leaves after 2 h of air exposure. The sensitive genotype (JL-3) showed wilting much earlier than the tolerant lines. Tolerant and sensitive genotypes severely wilted when exposed to air for 4 h, (Fig. 1b) and when returned into the nutrient solution and kept for 12 h, tolerant genotype (PDL-2) showed much faster recovery. On the other hand, JL-3 showed less recovery when returned to the nutrient solution (Fig. 1c).

### Variation in physiological and biochemical traits

#### Relative water content

**Fig. 2:**
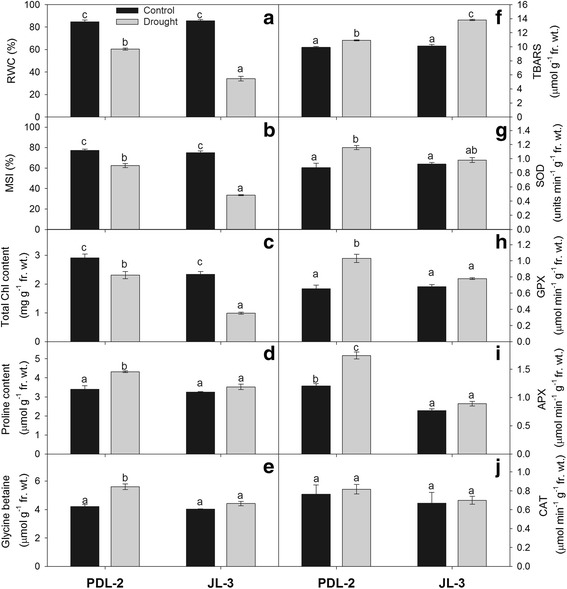
Changes in relative water content (**a**), MSI (**b**), total chlorophyll content (**c**) Proline content (**d**), Glycine betane (**e**), TBARS (**f**), SOD (**g**), GPX (**h**), APX (**i**), Catalase (**j**) of lentil genotypes (PDL-2 and JL-3) under control and drought stress. Bars with the same small letters do not statistically differ by the Tukey test at P ≤ 0.05

RWC data showed significant reduction under drought stress in tolerant and sensitive lentil genotypes. However, PDL-2 maintained RWC under drought stress showing a significantly lower reduction (28.6%) in RWC compared to JL-3 (60.1%) under drought stress (Fig. 2a).

#### Membrane stability index

Membrane stability index was reduced under drought stress, although PDL-2 had lower reduction in MSI with 19.3% reduction over the control as compared to JL-3 which showed quite higher reduction of 57.7% (Fig. 2b).

#### Chlorophyll content

Drought stress when imposed at the seedling stage significantly decreased chlorophyll contents in PDL-2 and JL-3. Though, PDL-2 showed lower reduction of chlorophyll contents (20.5%) than JL-3 (57.7%) (Fig. 2c).

#### Proline

**Fig. 3:**
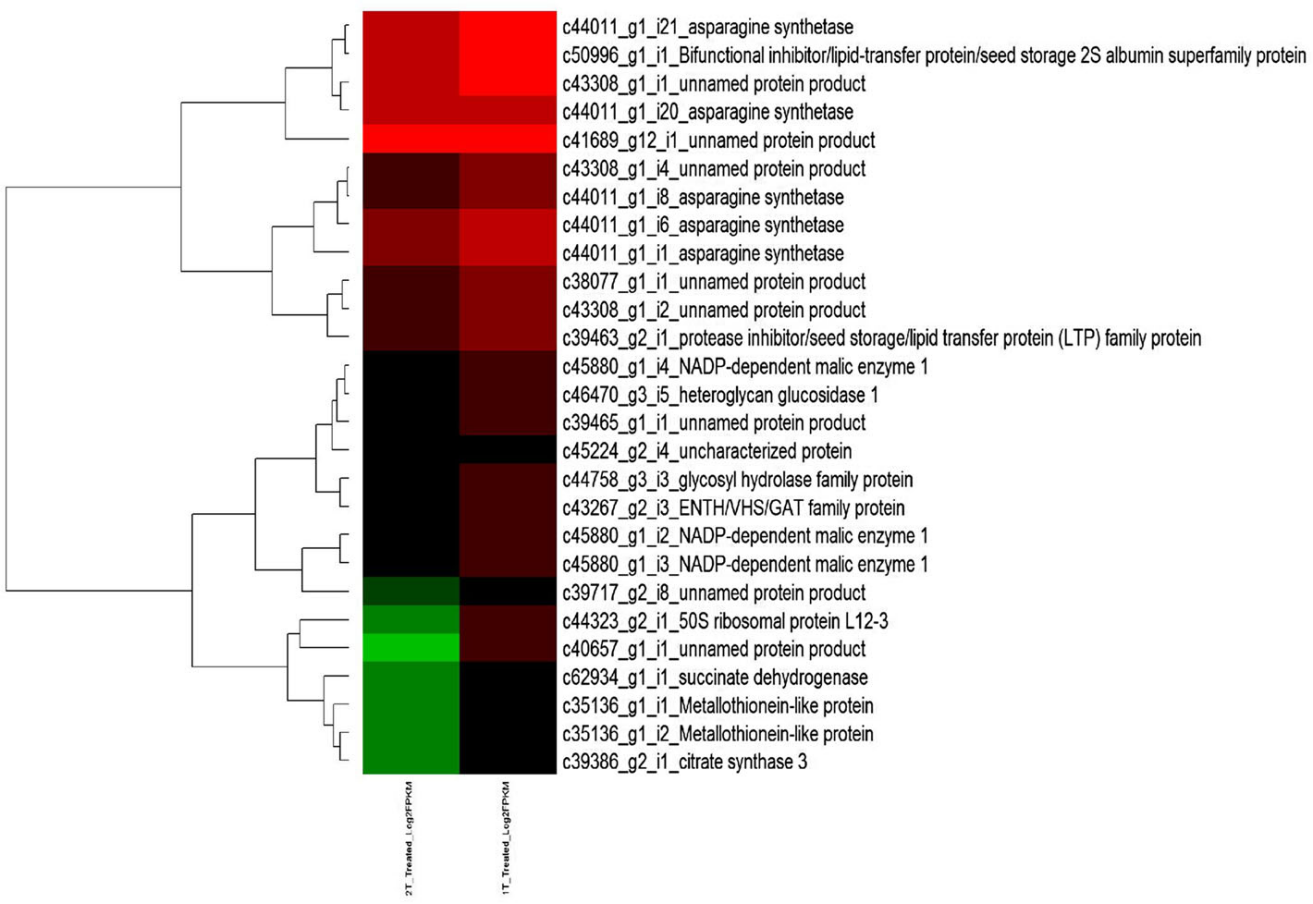
HeatMap of all Up regulated between samples with *p* value < 0.05 in 2T_Treated_1T_Treated

Under drought stress, there was observed significant increase in proline concentration of PDL-2 (27.2% over control). On the other hand, sensitive lentil genotype JL-3 didn’t show any significant change in proline accumulation (Fig. 3d).

#### Glycine betaine

In PDL-2, glycine betaine content increased significantly under drought with a 33.3% increase over the control, whereas in JL-3there was no significant change in its content (Fig. 2e).

#### Lipid peroxidation

Malondialdehyde (MDA) content was increased under drought stress in both the genotypes. However, the magnitude of its increment was prominent in JL-3 (36.5%) (Fig. 2f).

### Antioxidant activities

#### Catalase (CAT)

No significant differences in the activity of catalase enzyme was observed (Fig. 2j) under drought stress condition as compared to normal environment.

#### Superoxide dismutase (SOD)

There was higher SOD activity in the shoots of both tolerant and sensitive lentil genotypes under drought stress. SOD activity increased significantly in shoots of both the genotypes as compared to the control. The increase in SOD activity was higher in PDL-2 (32.5%) compared toJL-3 (6.1%) (Fig. 2g).

#### Ascorbate peroxidase (APX)

The activity of APX increased significantly under drought stress conditions in both tolerant and sensitive genotypes as compared to control. Seedlings grown under drought stress showed higher increase in APX activity in leaves of tolerant, PDL-2 genotype (44.8%) than sensitive genotype, JL-3 (15.9%) (Fig. 2i).

#### Glutathione peroxidase (GPX)

In drought conditions, GPX activity increased in the leaves of both the genotypes with a greater intensity in the leaves of PDL-2 (57.4%) than JL-3 (14.7%) (Fig. 2h).

### Total RNA integrity and cDNA library preparation

Total RNA was extracted from the both control and drought stressed plants and the quality of RNA was tested using Nanodrop and Agilent Bioanalyzer 2100. All the samples were satisfactory for library construction and sequencing with RIN value in between 6.7 and 7.9. Concentration of RNA samples (ng/μl) were 1823, 1493, 3111 and 1708 for 1C, 1 T, 2C and 2 T respectively. The TruSeq RNA fragmentation protocol for transcriptome analysis was performed on RNA after mRNA purification using elevated temperature. The fragmentation resulted in libraries with inserts ranging from 120 to 200 bp with a median size of 150 bp. To keep the selection consistent in subsequent stages, the fragments were eluted according to bead volume and incubation time.

### Whole transcriptome sequence data

Total paired-end reads of the samples PDL-2 (Control), PDL-2 (Treated), JL-3 (Control) and JL-3 (Treated) were 51435338, 56766344, 58518476 and 67764324, respectively. Clean bases were obtained by filtering impurities which yielded 23054590, 26212097, 27378757 and 34791064, respectively for above mentioned samples. Sequence analysis of control and drought treated cDNA libraries of PDL-2 and JL-3 genotypes produced 74032, 75500, 78328 and 81523 contigs, respectively. The maximum and minimum lengths of the contigs were 16502 bp and 201 bp, respectively (Table 2).

**Table 2:** Statistical analyses of transcriptome assembly obtained from drought-stress tolerant and sensitive genotypes

	CT (Control)		DT (Drought stress treatment)	
	1C	2C	1 T	2 T
Number of paired-end reads	51435338	58518476	56766344	67764324
Trimmed reads	23054590	27378757	26212097	34791064
Read Length (bp)	45806703	53918455	50000655	62520091
Total mapped	23054590 (95.23%)	27378757 (95.06%)	26212097 (95.06%)	34791064 (93.56%)
Q30 (%)	86.34	88.92	85.21	88.27
GC content (%)	41.18	41.36	41.25	42.66
Gene number	32247	33000	33375	33920
Total number of contigs	74032	75500	78328	81523
Maximum length of contigs	16502	16502	16502	16502
Minimum length of contigs	201	201	201	201
Average contigs length	1456.25	1457.83	1430.31	1402.86
Total length of contigs (bases)	107809785	110066495	112033681	114366089
N50	2011	2008	2000	1991
Number of proteins	52565	54613	54230	55667

### Analysis of differentially expressed genes

Differential gene expression of drought treated genotypes along with their controls revealed a total of 11,435 upregulated and 6,934 downregulated transcripts which were identified in the combinations of*.* 1C-1 T, 1C-2C, 1C-2 T, 1 T-2 T, 2C-1 T, 2C-2 T, where ‘1’ and ‘2’ represents drought tolerant (PDL-2) and drought sensitive (JL-3) genotypes respectively; ‘C’ represents control and ‘T’ as drought treated plants. Graphical representation of number of upregulated and downregulated transcripts in major comparative combinations is presented in Additional file 1: Figure S1. Data obtained from control and treated samples were subjected to combination wise comparison, from which DEGs were identified. A total of 6633 DEGs were identified (including 2919 solely expressed ones). Comparisons of total number of upregulated and downregulated contigs in all the genotypes are presented in Additional file 2: Figure S2 and Additional file 3: Figure S3), respectively in the form of Edward plots, which exhibited sharing of contigs between different combinations. When tolerant and sensitive genotypes were compared with their respective controls, 1514 and 1596 upregulated DEGs and 814 and 1012 downregulated DEGs were identified, respectively. When tolerant and sensitive genotypes were compared, 1417 upregulated and 1001 downregulated DEGs were identified. Upregulated DEGs constituted 65.03%, 61.20% and 58.60% in 1C-1 T, 2C-2 T and 1 T-2 T comparison groups, respectively. The results of significantly Differentially Expressed Genes obtained from DESeq & edgeR were compared and 45–55% of significantly expressing genes were found to be matching using both the software packages (edgeR & DESeq). When assembled transcripts were compared with NCBI non-redundant protein database using BLASTx, around 36% of transcripts were found to have confidence level of atleast 1e^-5^, where E-value < = 10^-5^ and similarity score > = 40% (Additional file 4: Figure S4a). Around 48% of assembled transcripts had similarity score of more than 60% at protein level with the existing proteins at NCBI database (Additional file 4: Figure S4b).

To describe the results, two different analysis methods were applied. Firstly, major DEGs whose expression differed significantly in different combination groups were identified based on the criteria that there *p*-value < 0.005 and log2_foldchange > 1 (Tables 3, 4, 5, 6, 7 and 8). When compared between sensitive and tolerant genotypes under drought stress condition, some of the DEGs whose expression was significantly upregulated belonged to Cyclin family (c44790_g4_i5, c44790_g4_i4, c44790_g4_i3, c44790_g4_i2), Aldehyde dehydrogenase family (c40657_g1_i1), PTR2/POT transporter family (c40074_g1_i3), Fatty acyl-CoA reductase family (c43159_g1_i4, c43159_g1_i1, c43159_g1_i8), Phosphatase 2A regulatory subunit B56 family (c36460_g1_i2) and the ones with downregulated expression belonged to Adaptor complexes small subunit family (c46344_g1_i1), c46344_g1_i1 (c45522_g2_i4, c45522_g2_i2), SHMT family (c45167_g2_i11), Cystinosin family (c43466_g1_i10), Polyglycohydrolase family (c45115_g1_i3), Cation transport ATPase (P-type) family (c42686_g1_i6), MenG/UbiE family (c44280_g1_i2) (Tables 5 and 6). In one of the comparison for 1 T-1C v/s 2 T-2C a total of 6720 genes were found significantly expressed with less than 0.05 FDR value (Additional file 5: Table S1).

**Table 3:** List of genes which are significantly upregulated in 1C vs 1 T

Contig_ID	Log2_Fold _change	*P*_value	1C FPKM	1 T FPKM	E-value	Protein families
c34613_g1_i1	4.383	2.37E-05	0.2	4.6	0	LDH family
c10084_g1_i1	3.202	0.000	2.1	20.7	7.00E-53	Peptidase C1 family
c40657_g1_i1	3.069	0.002	0.4	4.0	7.00E-109	Aldehyde dehydrogenase family
c42609_g1_i1	2.920	0.002	1.2	9.8	4.00E-142	Cation transport ATPase (P-type)
c10084_g2_i1	2.854	0.001	2.2	17.3	1.00E-119	Peptidase C1 family
c42609_g2_i2	2.776	0.001	1.2	9.8	0	Cation transport ATPase (P-type)
c44933_g1_i4	2.157	0.114	0.4	1.9	0	ABC transporter superfamily,Multidrug resistance exporter subfamily
c45817_g2_i1	2.087	0.061	1.3	6.1	7.00E-104	HSF family
c46246_g2_i6	2.029	0.073	6.2	27.0	3.00E-23	Glucose-6-phosphate dehydrogenase family
c43232_g1_i4	1.960	0.073	0.9	3.6	7.00E-169	Cytochrome P450 family
c29773_g1_i1	1.868	0.189	2.0	7.8	1.00E-101	Purine/pyrimidine phosphoribosyltransferase family
c54952_g1_i1	1.828	0.187	8.1	31.1	0	Peptidase C1 family
c39732_g1_i3	1.782	0.157	3.9	14.5	0	Ferrochelatase family
c44764_g6_i1	1.692	0.168	15.1	52.5	0	Heat shock protein 90 family
c39732_g1_i1	1.664	0.360	2.9	9.8	4.00E-148	Ferrochelatase family
c46066_g1_i4	1.660	0.280	8.1	27.6	3.00E-26	PA-phosphatase related phosphoesterase family

**Table 4:** List of genes which are significantly downregulated in 1C vs 1 T

Contig_ID	Log2_Fold_change	Adj *P*_value	1C FPKM	1 T FPKM	E-value	Protein families
c42732_g1_i1	-3.252	0.001	4.4	0.50	1.00E-108	NA
c35206_g1_i1	-2.466	0.025	2.9	0.57	0	Xanthine/uracil permease family
c44152_g1_i2	-2.252	0.034	3.4	0.77	0	Cyclic nucleotide-gated cation channel family
c44875_g1_i10	-1.977	0.106	5.0	1.37	0	MCM family
c41037_g1_i1	-1.863	0.241	1.6	0.46	0	ORC1 family
c46968_g1_i1	-1.671	0.254	13.4	4.53	0	Flavin monoamine oxidase family
c4763_g1_i1	-1.863	0.254	2.4	0.72	2.00E-180	Multi antimicrobial extrusion (MATE)family
c17950_g1_i1	-1.639	0.296	18.0	6.20	0	Fatty acid desaturase family

**Table 5:** List of genes which are significantly downregulated in 2 T vs 1 T

Contig_ID	Log2_Fold_change	Adj *P*_value	1 T FPKM	2 T FPKM	E-value	Protein families
c46344_g1_i1	-4.763	2.190E-05	0.1	4.1	3.00E-29	Adaptor complexes small subunit family
c45522_g2_i4	-7.607	3.750E-08	0.0	7.6	2.00E-28	MCM family
c45522_g2_i2	-4.848	3.830E-05	0.2	7.2	1.00E-28	MCM family
c45167_g2_i11	-4.284	1.530E-09	1.9	39.0	1.00E-55	SHMT family
c43674_g8_i12	-5.763	2.168E-04	0.1	5.4	4.00E-63	NA
c40173_g1_i2	-3.800	1.737E-04	1.0	14.8	3.00E-11	NA
c30999_g1_i3	-2.521	7.391E-03	2.2	13.1	4.00E-70	NA
c43466_g1_i10	-2.715	8.091E-03	1.4	9.6	3.00E-47	Cystinosin family
c30999_g1_i2	-2.385	1.423E-02	2.4	13.5	2.00E-70	NA
c30999_g1_i1	-2.356	1.761E-02	2.3	12.4	3.00E-70	NA
c45115_g1_i3	-2.245	6.781E-02	0.8	4.2	2.00E-126	Poly(ADP-ribose) glycohydrolase family
c46402_g1_i7	-1.962	8.605E-02	6.4	26.1	2.00E-20	NA
c45311_g8_i1	-1.591	1.056E-01	19.7	62.6	5.00E-82	NA
c42686_g1_i6	-2.031	1.176E-01	2.6	11.3	9.00E-67	Cation transport ATPase (P-type) family,
c44280_g1_i2	-2.252	1.641E-01	1.0	5.0	1.00E-98	Class I-like SAM-binding methyltransferase superfamily, MenG/UbiE family

**Table 6:** List of genes which are significantly upregulated in 2 T vs 1 T

Contig_ID	Log2_Fold_change	Adj *P*_value	1 T FPKM	2 T FPKM	E-value	Protein families
c44790_g4_i5	3.857	0.000	6.5	0.5	2.00E-21	Cyclin family, Cyclin AB subfamily
c44790_g4_i4	5.265	0.000	7.6	0.2	3.00E-12	Cyclin family, Cyclin AB subfamily
c44790_g4_i3	4.680	0.000	5.8	0.2	6.00E-29	Cyclin family, Cyclin AB subfamily
c44790_g4_i2	4.573	0.000	6.6	0.3	2.00E-21	Cyclin family, Cyclin AB subfamily
c40657_g1_i1	7.966	0.000	4.0	0.0	7.00E-109	Aldehyde dehydrogenase family
c40074_g1_i3	2.617	0.008	5.1	0.9	0	PTR2/POT transporter family
c43159_g1_i4	3.120	0.011	3.4	0.4	9.00E-170	Fatty acyl-CoA reductase family
c45964_g1_i5	2.932	0.018	7.9	1.1	4.00E-25	NA
c36460_g1_i2	2.017	0.049	6.8	1.8	2.00E-85	Phosphatase 2A regulatory subunit B56 family
c43159_g1_i1	2.943	0.051	3.0	0.4	8.00E-146	Fatty acyl-CoA reductase family
c43159_g1_i8	2.344	0.080	3.5	0.7	4.00E-65	Fatty acyl-CoA reductase family
c30244_g1_i2	1.766	0.165	7.7	2.4	2.00E-113	NA
c33568_g1_i1	1.599	0.214	15.2	5.3	7.00E-89	Cyclophilin-type PPIase family

**Table 7:** List of genes which are significantly downregulated in 2C vs 2 T

Contig_ID	Log2_Fold_change	Adj *P*_value	2C FPKM	2 T FPKM	E-value	Protein families
c32642_g1_i1	-3.614	0.0009	3.1	0.3	0	Glycosyltransferase 8 family, Glycogenin subfamily
c38916_g2_i3	-3.232	0.0097	2.9	0.3	1.00E-83	Cyclin family, Cyclin AB subfamily
c44152_g1_i2	-2.667	0.0177	3.8	0.6	0	Cyclic nucleotide-gated cation channel family
c37946_g1_i2	-2.916	0.0244	4.1	0.6	1.00E-132	MAD2 family
c43673_g1_i5	-2.574	0.0313	4.8	0.8	0	Cytochrome P450 family
c44152_g1_i1	-2.545	0.0818	3.1	0.6	0	Cyclic nucleotide-gated cation channel family
c24995_g1_i1	-2.173	0.1794	2.8	0.7	0	WD repeat CDC20/Fizzy family
c25920_g1_i1	-2.094	0.2470	3.7	0.9	6.00E-162	NA
c33730_g1_i3	-1.879	0.3855	2.3	0.7	6.00E-96	HSF family, Class A subfamily

**Table 8:** List of genes which are significantly upregulated in 2C vs 2 T

Contig_ID	Log2_Fold_change	*P*_value	2C FPKM	2 T FPKM	E-value	Protein families
c34613_g1_i1	3.597	0.000	0.7	8.8	0	LDH/MDH superfamily, LDH family
c38367_g1_i1	2.564	0.026	2.8	17.8	0	Phosphofructokinase type A (PFKA) family,
c43958_g2_i3	2.615	0.060	0.7	4.6	2.00E-157	NA
c11279_g1_i1	2.600	0.073	3.7	24.0	0	Cation transport ATPase (P-type) family,
c33686_g1_i1	2.757	0.084	0.3	2.3	6.00E-112	ELO family
c43958_g2_i2	2.312	0.190	0.6	2.9	6.00E-134	NA
c44933_g1_i4	1.904	0.239	1.7	6.6	0	ABC transporter superfamily, ABCB family, Multidrug resistance exporter subfamily
c45817_g2_i1	1.875	0.262	2.3	8.7	7.00E-104	HSF family, Class A subfamily
c45088_g1_i11	1.907	0.283	2.2	8.7	5.00E-120	Carbon-nitrogen hydrolase superfamily, BUP family
c18989_g1_i3	1.811	0.314	1.5	5.4	0	ABC transporter superfamily, ABCB family, Multidrug resistance exporter subfamily
c41595_g1_i2	2.233	0.331	0.4	2.1	1.00E-168	NA
c44789_g2_i5	1.783	0.346	3.4	12.3	1.00E-07	Organic cation transporter family

Secondly, major DEGs related to drought stress response and regulations were also analyzed separately (Table 9 and Additional file 6: Figure S5). Hierarchical heat map for drought related DEGs were generated for all the combinations. Top up-regulated DEGs in drought stressed tolerant genotypes when compared to their controls were that of delta 1-pyrroline-5-carboxylate synthase 2 (c46123_g1_i3, c46123_g1_i16, c46123_g1_i7, c46123_g1_i11, c46123_g1_i4, c46123_g1_i8), uncharacterized protein (c20592_g1_i1, c36412_g1_i1, c36412_g1_i2, c36412_g1_i3, c20592_g1_i2), unnamed protein product (c32354_g1_i1, c41157_g14_i4), 50S ribosomal protein L12-3 (c44323_g2_i1), Succinyl-CoA ligase [GDP-forming] subunit alpha-2 (c30331_g1_i1), Ninja-family protein AFP3 (c37153_g1_i1), peroxidase 52 (c42356_g1_i2, c42356_g1_i4, c42356_g1_i6), Fe superoxide dismutase 2 (c17843_g1_i1), CLP protease regulatory subunit X (c8801_g1_i1), major facilitator protein (c37965_g1_i1), cytochrome P450, family 81, subfamily D, polypeptide 8 (c40368_g1_i1), pathogenesis-related 4 (c43084_g1_i3), R2R3 family MYB transcription factor (c36353_g1_i4), succinate dehydrogenase [ubiquinone] flavoprotein subunit 1 (c62934_g1_i1), electron transfer flavoprotein subunit beta (c27176_g1_i1), polygalacturonase inhibitor 2 (c39114_g3_i1) and Metallothionein-like protein (c35136_g1_i2) (Additional file 7: Figure S6). Top downregulated ones included unnamed protein product (c24944_g2_i1, c31105_g1_i1), peroxidase (c12575_g1_i1), bifunctional inhibitor/lipid-transfer protein/seed storage 2S albumin superfamily protein (c8386_g1_i1), sigma factor binding protein 1 (c8472_g1_i1), stress responsive A/B Barrel domain-containing protein (c63112_g1_i1), aquaporin PIP1-3 (c45602_g6_i1), abscisic acid receptor PYL6 (c30837_g1_i2) and probable xyloglucanendo transglucosylase/hydrolase protein 33 (c29493_g1_i5) (Additional file 8: Figure S7).

**Table 9:** Drought related DEGs in different combinations

Drought related DEGs	1C *vs.* 1 T	1 T *vs.* 2 T	2C *vs.* 2 T
Total DEGs	410	368	400
Up-regulated DEGs	311	213	270
Down-regulated DEGs	99	155	130

**Fig. 4:**
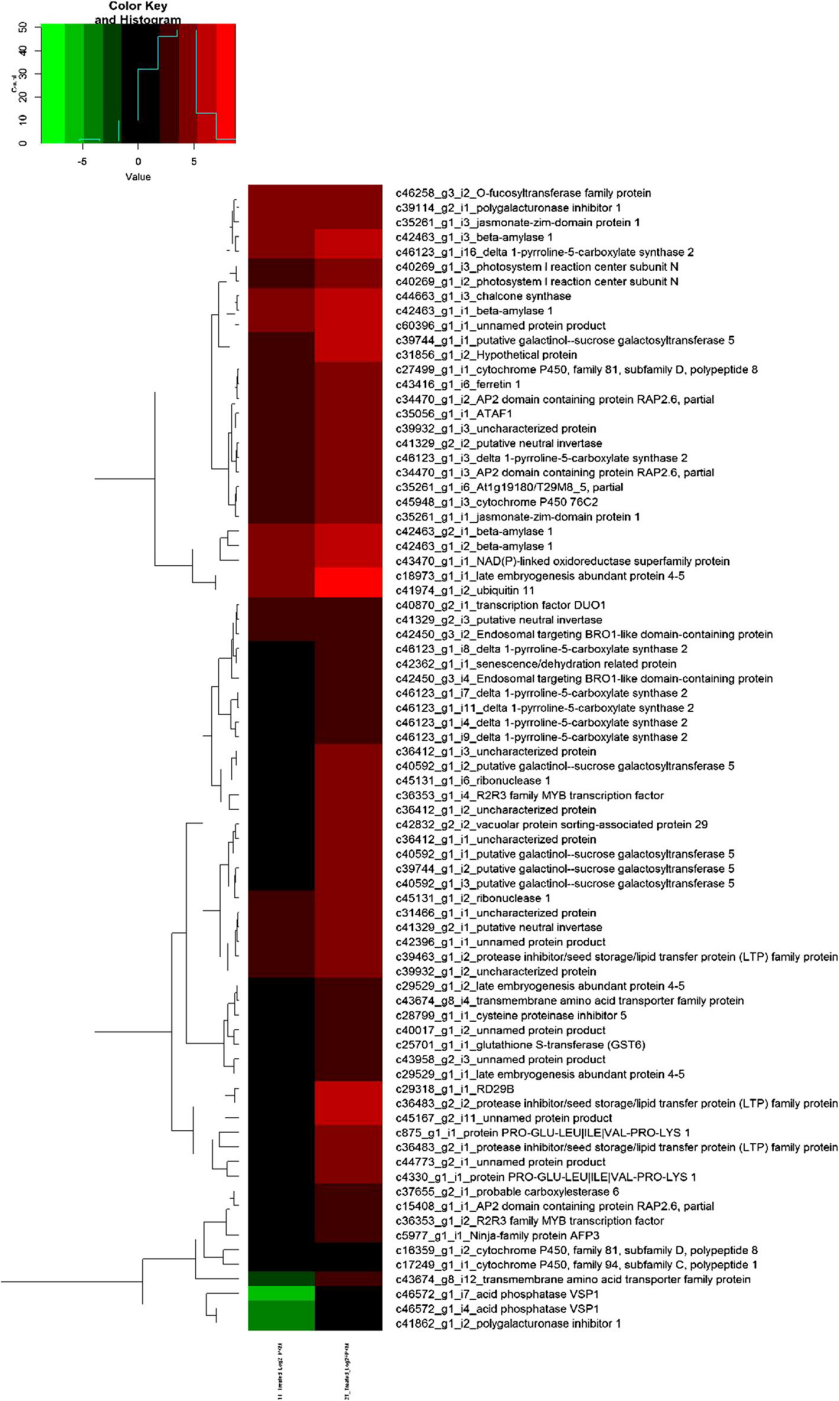
HeatMap of all Down regulated between samples with *p* value < 0.05 in 2T_Treated_1T_Treated

Most significantly up-regulated DEGs in tolerant genotypes with log 2 foldchange > 3 when compared to sensitive ones under stress conditions were that of unnamed protein product (c40657_g1_i1, c39717_g2_i8), 50S ribosomal protein L12-3 (c44323_g2_i1), citrate synthase 3 (c39386_g2_i1), succinate dehydrogenase [ubiquinone] flavoprotein subunit 1 (c62934_g1_i1), Metallothionein-like protein (c35136_g1_i2, c35136_g1_i1) and NADP-dependent malic enzyme 1 (c45880_g1_i3, c45880_g1_i2) (Fig. 3). The most significantly downregulated ones in tolerant genotypes included acid phosphatase VSP1 (c46572_g1_i7, c46572_g1_i4), unnamed protein product (c44773_g2_i1, c45167_g2_i11), transmembrane amino acid transporter family protein (c43674_g8_i12), protein PRO-GLU-LEU|ILE|VAL-PRO-LYS 1 (c875_g1_i1, c4330_g1_i1), polygalacturonase inhibitor 1 (c41862_g1_i2), protease inhibitor/seed storage/lipid transfer protein (LTP) family protein (c36483_g2_i2, c36483_g2_i1), RD29B (c29318_g1_i1), Ninja-family protein AFP3 (c5977_g1_i1), AP2 domain containing protein RAP2.6, partial (c15408_g1_i1), late embryogenesis abundant protein 4-5 (-3.483159), R2R3 family MYB transcription factor (c36353_g1_i4, c36353_g1_i2) and probable carboxylesterase 6 (c37655_g2_i1) (Fig. 4).

When sensitive genotypes exposed under drought stress were compared to control, the genes which were found to be upregulated included delta 1-pyrroline-5-carboxylate synthase 2 (c46123_g1_i3, c46123_g1_i16, c46123_g1_i7, c46123_g1_i11, c46123_g1_i4, c46123_g1_i9, c46123_g1_i8), RD29B (c29318_g1_i1), R2R3 family MYB transcription factor (c36353_g1_i4), uncharacterized protein (c36412_g1_i1, c36412_g1_i2, c36412_g1_i3, c38711_g2_i2), short-chain dehydrogenase reductase 5 (c41164_g1_i1), galactinol synthase 1 (c18069_g1_i1), HXXXD-type acyl-transferase-like protein (c61853_g1_i1), PR-6 proteinase inhibitor family protein (c24138_g1_i1), putative galactinol--sucrose galactosyltransferase 5 (c39744_g1_i1, c40592_g1_i3, c40592_g1_i1), unnamed protein product (c41157_g14_i4), protein MILDEW RESISTANCE LOCUS O 12 (c22448_g1_i1), protease inhibitor/seed storage/lipid transfer protein (LTP) family protein (c36483_g2_i2, c36483_g2_i1), late embryogenesis abundant protein 4-5 (c18973_g1_i1) and short-chain dehydrogenase reductase 5 (c41164_g1_i2) (Additional file 9: Figure S8) The genes which were downregulated in sensitive genotypes when compared to their control included protease inhibitor/seed storage/lipid transfer protein (LTP) family protein (c9740_g1_i1), probable xyloglucanendo transglucosylase/hydrolase protein 33 (c29493_g1_i1, c29493_g1_i5, c29493_g1_i3, c29493_g1_i4, c29493_g1_i6, c29493_g1_i2) and unnamed protein product (c24944_g2_i1, c58927_g1_i1, c41529_g3_i1, c60003_g1_i1, c11737_g1_i1) (Additional file 10: Figure S9).

### Validation through qRT PCR

**Fig. 5:**
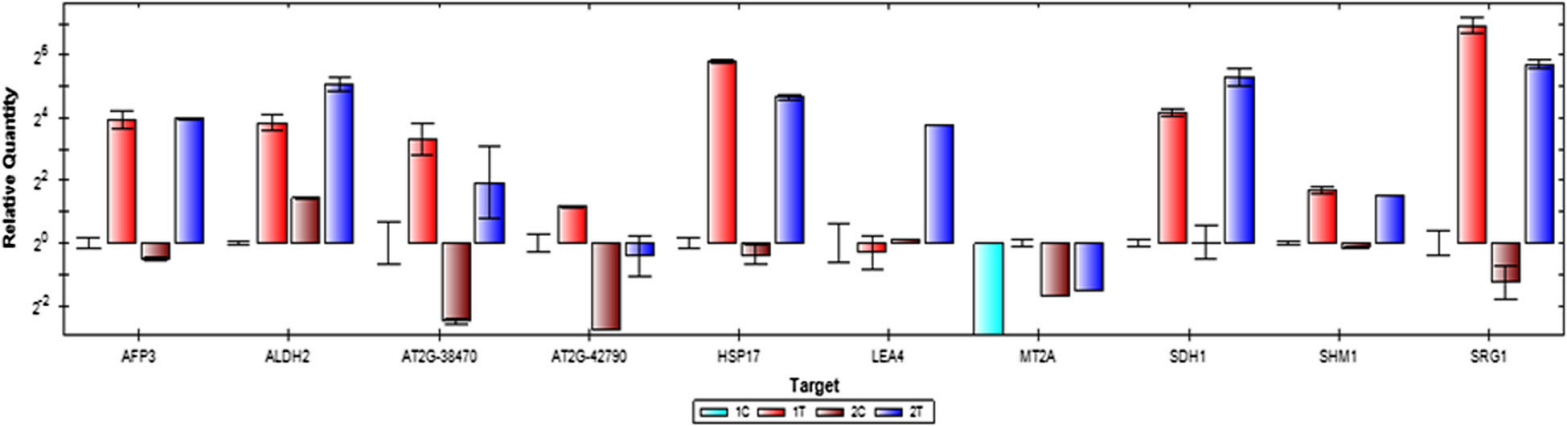
Relative expression profile of genes by real time PCR. Relative quantification was obtained through 2^-(ΔΔCT)^ method using β-tubulin as reference gene. Data represent the average from four biological replicates and the error bars indicate the standard deviation (± SD)

To validate the data, ten DEGs with log2fold change above 4 (ALDH2B4 ALDH2 At3g48000 T17F15.130, At2g42790, MT2A At3g09390 F3L24.28, SRG1 At1g17020 F20D23.28 F6I1.30, HSP17.6B At2g29500 F16P2.12, LEA4-5 At5g06760, SDH1-1 At5g66760 MSN2.16, SHMT1 STM At4g37930 F20D10.50, AFP3 At3g29575 MWE13.5 and At2g38470) were selected for RT- PCR analysis. The level of expression of the genes amplified using real time PCR has been shown in Fig. 5. Raw data were log2 transformed and compared to transcriptomics data, showed a close relationship and validation of differential expression of the genes under drought stress conditions.

### Functional classification of DEGs

For functional classification of DEGs and to investigate the metabolic pathways in which they are involved for their fight against drought, the GO terms for transcripts were extracted and KEGG pathway annotation analysis was performed on the identified DEGs. A total of 413 GO annotation terms were extracted where 176 were within molecular process, 128 in cellular and 109 in biological process groups (Additional file 11: Figure S10).

**Fig. 6:**
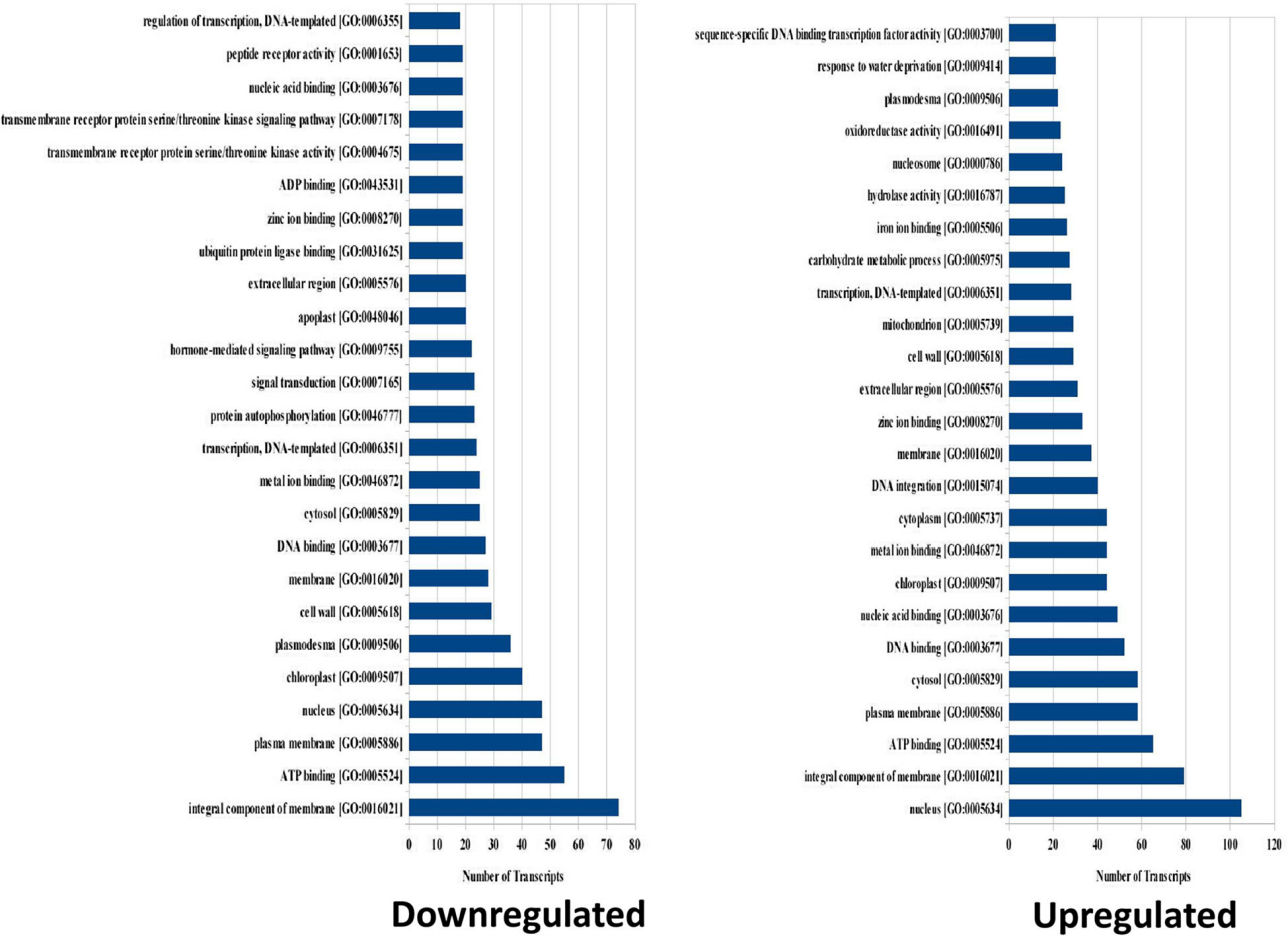
Top 25 GO terms for down-regulated and up-regulated transcripts in ‘2 T’ as compared to ‘1 T’

When drought tolerant plants were compared to control, the top three significantly enriched GO annotation categories for downregulated DEGs were nucleus (GO:0005634), integral component of membrane (GO:0016021) and plasma membrane (GO:0005886). Apart from last two (GO:0016021, GO:0005886), third significantly enriched GO annotation for upregulated DEGs in tolerant plants was ATP binding (GO:0005524) (Additional file 12: Figure S11). GO annotation in sensitive genotypes revealed that categories similar to tolerant ones were found in sensitive genotypes also. When sensitive genotypes were compared with their controls, highly enriched GO annotation categories were integral component of membrane (GO:001602), plasma membrane (GO:0005886) and nucleus (GO:0005634) in downregulated transcripts along with GO terms GO:0016021, GO:0005886 and GO:0005634 in upregulated transcripts (Additional file 13: Figure S12). Also, when sensitive and tolerant genotypes were compared, 3 most significant GO categories were integral component of membrane (GO:0016021), ATP binding (GO:0005524) and plasma membrane (GO:0005886) in downregulated transcripts and nucleus (GO:0005634), integral component of membrane (GO:0016021) and ATP binding (GO:0005524) in upregulated transcripts (Fig. 6).

The pathway annotation analysis of a total of top 202 genes in the three comparison groups *viz.* 1C vs 1 T, 2C vs 2 T and 2 T vs 1 T revealed 28 unique drought related DEGs annotated to KEGG pathways including 42 metabolic pathways (Table 10)

**Table 10:** KEGG pathway annotation and number of DEGs in different comparison groups

KEGG pathway item	DEGs number
Metabolic pathways	15
Biosynthesis of secondary metabolism	13
Carbon metabolism	6
Citrate cycle	4
Protein processing in endoplasmic reticulum	4
Biosynthesis of amino acids	3
Phenylpropanoid biosynthesis	3
Galactose metabolism	3
Arginine and proline metabolism	2
Limonene and pinene metabolism	2
Plant hormone signal transduction	2
Lysine degradation	2
Pyruvate metabolism	2
Glyoxylate and dicarboxylate metabolism	2
Ribosome	1
Propanoate metabolism	1
Peroxisome	1
Stilbenoid, diarylhepatoid and gingerol biosynthesis	1
Oxidative phosphorylation	1
Carotenoid biosynthesis	1
Glutathione metabolism	1
Phenylalanine metabolism	1
Fatty acid degradation	1
Pentose and glucuronateinterconversions	1
Glycerolipid metabolism	1
Beta-Alanine metabolism	1
Valine, leucine and isoleucine degradation	1
Ascorbate and aldrate metabolism	1
Tryptophan metabolism	1
Histidine metabolism	1
Diterpenoid biosynthesis	1
Endocytosis	1
Spliceosome	1
2-oxocarboxylic acid metabolism	1
Plant-pathogen interaction	1
Starch and sucrose metabolism	1
Alanine, aspartate and glutamate metabolism	1
Carbon fixation in photosynthetic organisms	1
Glycine, serine and threonine metabolism	1
Cyanoamino acid metabolism	1
One carbon pool by folate	1

The most frequently associated pathways were metabolic pathways (15), followed by biosynthesis of secondary metabolites (13), carbon metabolism (6), citrate cycle and protein processing in endoplasmic reticulum (4 each). Some of the DEGs like AT3G55610 for arginine and proline metabolism; AT5G40390 for galactose metabolism were upregulated in both 1C vs 1 T and 2C vs 2 T comparison groups. Similarly, DEGs like AT3G27850 for propanoate metabolism; AT4G37370 for stilbenoid, diarylhetanoid, gingerol biosynthesis and limonene and pinene degradation; AT5G66760 for oxidative phosphorylation; AT3G48000 for fatty acid degradation and beta-Alanine metabolism etc. were upregulated in both 1C vs 1 T and 2 T vs 1 T comparison groups.

Some drought responsive transcription factors that bind to specific DNA sequences and control the rate of transcription were also identified. TFs are the key entities in transduction of stress related signals. When compared in 1C-1 T, 1 T-2 T and 2C-2 T comparison groups most of transcription factors belonged to TF families like AP2/ERF family (subfamily ERF or RAV), WRKY group II b and III families, HD-ZIP homeobox family or BZIP family of transcription factors. Also, several SSRs (9949), SNPs (8260) and INDELs (1248) were identified which could be further developed and used for drought related studies in lentil and other crops using Samtools mpileup toolkit. SNP calling analysis was performed by GATK toolkit, Haplotype caller tool (version 3.6-0) using default parameters (Additional file 14: Table S2).

## Discussion

### Plant water stress

Hydroponic is most effective and practical approach for screening large number of genotypes in small area because it is easy in handling and has possibility of better controlled environment. Another advantage is that it is non destructive and plants can be screened at a early stage of growth, and tolerant plants can be selected and transferred to pots or field for further assessment of drought tolerance at subsequent stages of growth [19, 20]. In the hydroponic technique, when plants were exposed to air for 4 h, all of them initially get wilted (Fig. 1). But the marked difference occurred when they were returned to the nutrient solution for 12 h, only the tolerant genotype (PDL-2) showed strong recovery, whereas the sensitive genotype (JL-3) showed less recovery. This suggested that even 4 h exposure to stress resulted in completely affected plant metabolism in the sensitive genotype, which was not observed in drought tolerant genotype. The exposure to air did not affect the basic metabolic activities of the plants and they retained the capacity to revive back to normal life when water became available [20]. The visual observations of wilting after relatively short durations of air exposure provided a suitable and reliable ranking of genotypes under long-term and/or more severe drought stress conditions. Therefore, it suggests that visual assessment of plant wilting and seedling survivability may offer suitable parameters for quick characterization of drought tolerance even at seedling stage.

### Physiological and biochemical attributes

Plant RWC decreased significantly in both lentil genotypes under drought stress but PDL-2 maintained significantly higher RWC in both the control and water stressed conditions (Fig. 2a). It shows that the higher RWC enabled the tolerant lentil genotype to perform better in terms of physio-biochemical processes under water stress conditions. Tolerant genotype (PDL-2) produced higher stable yield, probably because water retention ability in plant is one of the components of tolerance mechanisms [19]. Higher RWC has been reported to be associated with higher level of photosynthetic pigments, membrane stability index, osmolytes and antioxidant activities in maize [25]. Opening of stomata results in more transpiration and subsequently reduction in RWC. Under these conditions, the genotype loses more water, particularly if drought is prolonged, plant recovery is impossible and ultimately it may die. Tolerant genotypes can maintain higher RWC in their leaves through stomatal closure and consequently reduction in leaf gas exchange [26]. The RWC in leaves of drought stressed cultivars decreased significantly. Many researchers have reported large reductions in relative water content and water potential in the leaves under drought stress [27–31].

Drought stress caused a strong loss of photosynthetic pigments although; PDL-2 had higher chlorophyll contents than JL-3 under water stress. Contrasting effects of drought stress on the plant pigments have been reported previously [32, 33]. Similarly drought-tolerant genotypes have been reported to maintain higher chlorophyll content than sensitive ones [34].

The synthesis of osmolytes including proline and glycine betaine is widely reported in plants to stabilize membranes and maintain the conformation of proteins at low leaf water potential. But variability in synthesis as well as accumulation of osmolytes occurs among intra and inter plant species. Our results showed that lentil genotypes which performed better under water deficit conditions had higher levels of proline and glycine betaine content as compared to those found sensitive to stress conditions (Fig. 2d and e). Higher proline concentration has been reported to be involved in reducing the photo damage to chloroplast thylakoid membranes by scavenging and/or reducing the production of ROS [35–37]. Therefore, higher concentration of proline has been suggested as one of the parameters for selection for stress tolerant plant [38]. Similarly, proline has also been reported to protect and stabilize ROS scavenging enzymes and activate alternative detoxification pathways in plants subjected to various abiotic stresses [39]. Therefore, higher proline may act as a direct antioxidant as well as an activator of antioxidant mechanisms. Accumulation of proline has been associated with drought stress avoidance in maize, wheat and chickpea also [34, 40, 41]. Glycine betaine (GB) is another effective compatible solute which increases in the chloroplast of plants, when exposed to environmental stresses [42]. An increased accumulation of glycine betaine content was noticed in tolerant genotypes. Higher level of GB under drought stress has been reported in many plants like barley [43, 44].

MDA has been suggested as a marker of oxidative stress-induced lipid injury and its concentration varies in response to abiotic stresses [45, 46]. Lipid peroxidation, in turn, is an indicator of the prevalence of free radical reactions occurring in tissues and indicates a relationship between drought and oxidative stress [47, 48]. The genotypes show better performance under water deficit conditions have been observed to have lower levels of MDA content in the roots (Fig. 2f) thus they protect themselves from lipid peroxidation of membrane systems as compared to the genotypes which had higher levels of MDA content. The similar results were observed in wheat and fababean under drought stress conditions [49, 50].

Antioxidant enzymes like CAT and POX are key enzymes in scavenging and detoxification of hydrogen peroxide, a hazardous by product of photorespiration [51]. In this study the activity of these enzymes was induced due to drought stress. The increase in activity was higher in POX across genotypes responding to stress. Similar induced activity of above enzymes under stress conditions has been reported previously [51, 52]. Highest activity of CAT in response to drought was observed in PDL-2 while lowest POX activity under stress condition was recorded in JL-3. This is clear that these genotypes experienced oxidative stress and their antioxidant enzymes triggered to detoxify cells. These results are in consistence earlier reports showing higher antioxidant enzyme activity in wheat cultivars [53, 54].

### Transcriptome analysis and stress responsive genes

Transcriptome sequences data can be a valuable resources especially for the species without a completely sequenced genome like lentil. In lentil drought stress mediated gene expression has been sparsely studied. In transcriptome analysis, N50 is an important criterion to decide the quality of assembly and with a N50 value of approximately 2000, quality of assembly was found to be very high [55–57]. A total of 11,435 upregulated and 6,934 downregulated transcripts were identified through differential gene expression of drought treated genotypes and their controls. Gene expression analysis revealed that genes involved in oxidation-reduction process, correct folding of protein, TCA cycle, electron transport chain, organ senescence and reduction of stomatal conductance are more severely upregulated in drought tolerant genotypes than the sensitive ones, whereas genes for transcription binding, GABA synthesis, synthesis of cell wall protein, those involved in negative regulation of absicisic acid etc. are downregulated in tolerant genotype as compared to sensitive ones. In tolerant genotype, the activity of unnamed protein product (NCBI accession no. 291047692, patented by Journal No. WO 2010020654-A2 25-FEB-2010) which belonged to aldehyde dehydrogenase family was most significantly upregulated with a log2 fold change of 7.9. Water stress often results in concentration of reactive toxic molecules like aldehydes, which can cause lipid peroxidation and alteration in proteins and nucleic acids. The aldehyde dehydrogenase family is a large family of enzymes which are regarded as “aldehyde scavengers” and irreversibly convert these aldehydes into acids which will result in less damage caused by several abiotic stresses including drought [58].

Other significantly upregulated DEGs within a log2 fold change of 3 to 6 belonged to 50S ribosomal protein L12-3, citrate synthase 3, succinate dehydrogenase [ubiquinone] flavoprotein subunit 1, Metallothionein-like protein and NADP-dependent malic enzyme 1. 50S ribosomal protein L12-3 is a chloroplast precursor and upregulation of its protein may represent the tolerant cultivar attempt to protect and sustain the correct folding of other protein in addition to accelerated degradation of unfolded/incorrectly folded or stress damaged protein [59]. Citrate synthase 3 is peroxisomal citrate synthase which is required for fatty acid respiration in seedlings where citrate is exported from peroxisomes into mitochondria during respiration of triacylglycerol [60]. Succinate dehydrogenase [ubiquinone] flavoprotein subunit 1 is located in mitochondrial respiratory chain complex II and is involved in mitochondrial electron transport. It was upregulated in Ilex paraguariensis leaves in response to water deficit and abscisic acid [61]. Metallothionein-like proteins are low molecular weight, cysteine-rich, soluble, and metal-binding proteins which are found in both plant and animal tissues. Involvement of these proteins in drought stress has also been confirmed in peanut [62, 63]. Gorantla et al. found that metallothionein-like proteins represented the most abundant group of drought stressed transcripts in rice cultivar (Nagina 22) which helped in metal detoxification [64]. Similarly, two genes encoding metallothionein-like proteins were identified which were induced under drought stress in *B. napus* suggesting function of metallothionein-like proteins in drought stress [65]. NADP-dependent malic enzyme 1 plays an important role as anti-drought. The majority of water loss from plants occurs through stomata. When stomata are open, the concentration of potassium chloride and/or malate is high in guard cells, which enhance their turgor pressure and results in increased pore size. These are widely distributed in plant, which mainly appear in mitochondria, chloroplast as well as cytoplasm and catalyze the oxidative decarboxylation of malate to produce pyruvate, CO_2_ and NADPH under metallic ions [66]. When leaf stoma are closed under drought stress, the malate concentration in cell decreases and NADP-ME activity increases [67].

In this study, it was found that under drought stressed condition for tolerant as well as sensitive genotypes there is higher percentage of upregulated DEGs. Further, some of the DEGs involved in TCA cycle, respiratory electron transport chain, ion channel transport, ABC family protein mediated transport, HSFs activation, metabolism of glucose are upregulated when tolerant and sensitive genotypes are compared with their controls whereas it has been reported that expression of genes involved in photosynthesis, photorespiration and carbohydrate metabolism were more drastically downregulated in drought tolerant genotypes than the sensitive ones [68].

### Pathway analysis for drought tolerance

The detailed classification of KEGG pathways for DEGs with 3–7 log2fold change fall under 42 pathways, where a significant number of DEGs belonged to secondary metabolism category including stilbenoid, diaryl heptanoid and gingerol biosynthesis, phenylpropanoid biosynthesis, diterpenoid biosynthesis along with that of carbon metabolism and citrate cycle; protein processing in endoplasmic reticulum, biosynthesis of amino acids, galactose metabolism, plant hormone signal transduction, nitrogen metabolism including alanine, aspartate and glutamate metabolism, fatty acid degradation etc. Similar results have been reported in *Ammopiptanthus mongolicus* leaves under drought stress [69].

Antioxidant enzymes constitute “first line of defence” against ROS generated during environmental stresses like drought [70]. Superoxide dismutase is one of the major classes of antioxidants that catalyzes the first step in ROS scavenging system and removes superoxide radicals by converting them into H_2_O_2_ and O_2_ [71]_._ Higher SOD activity in drought tolerant lentil genotype, PDL-2 may be one of the mechanisms for drought tolerance as AT5G51100 gene was highly upregulated in tolerant genotype under drought stress, which is involved in hydrogen peroxide metabolism in peroxisomes. The production of H_2_O_2_ can directly be countered by the activities of catalase and nonspecific peroxidise. Phenylpropanoids constitute a secondary antioxidant system, and activated upon due to depletion of primary antioxidant defences and control cellular H_2_O_2_ within a sub-lethal concentration range [70]. Peroxidase 52 upregulated in this study are involved in biosynthesis of phenylpropanoids like p-Hydroxy phenyl lignin, Guaiacyl lignin and Syringyl lignin, which must have played a major role in ROS scavenging. Phenyl ammonia lyase activity which is a key enzyme involved in biosynthesis of isoprenoid antioxidant compounds was found to increase sharply in tolerant genotype under drought stress condition. Similar results have been reported in maize inbreds [72]. Biosynthesis of other secondary metabolities like stilbenoid, diaryl hepatanoid and gingerol were also found to increase due to upregulation of gene encoding cytochrome P450, polypeptide 8, which is involved in conversion of resveratrol to piceatanol, a crucial step involved in synthesis of these metabolites.

Stress-inducible galactinol-synthase, a member of glycosyl transferases family plays a key role in enhancing level of galactinol and raffinose under abiotic stress conditions, which is important osmoprotectants for drought stress tolerance [73]. Galactinol synthase is involved to transfer of UDP-D galactose to myo-inositol and is considered the main regulator of this biosynthetic pathway [74]. In this study, galactinol synthase 1 gene has been upregulated many folds in both 1C vs 1 T and 2C vs 2 T comparison groups suggesting its role in regulation of drought tolerance in lentil. Several studies have shown that the expression of galactose synthase gene is involved in response to several abiotic stress tolerance mechanisms [74–77].

Under drought stress conditions, the endoplasmic reticulum (ER) protein folding machinery reaches a limit and the demand for protein folding exceeds its capacity. Therefore unfolded or miss-folded proteins increase in the ER, and trigger an unfolded protein response. This results in up regulating the expression of genes encoding components of protein folding machinery or the ER- associated degradation system [78]. The same has been visualized in lentil as the expression of genes encoding heat shock protein 70, which is part of ubiquitin ligase complex and ER- associated degradation system and 70B, which is characterized as molecular chaperone and that of heat shock protein 22 (shsp) were significantly upregulated.

Gene encoding Glutathione S-transferase TAU 20 (AT1G78370), which is involved in glutathione metabolism, was upregulated in tolerant genotype under drought condition. Glutathione S-transferases are involved in protection under various stress conditions by detoxifying endogenous plant toxins which increases under oxidative stress [79]. GSTs play a major role under drought conditions by conjugation of glutathione with electrophilic substrates to increase their solubility and facilitating further metabolic processing [80]. There are reports, which suggest involvement of GSTs in drought stress [81, 82]. AT1G78370, the gene upregulated in this study, has been shown to physically interact with Far-Red Insensitive 219 (FIN219) in response to light and play a crucial role in cell elongation and plant development [83].

Xyloglucanendo transglucosylase/hydrolase is important enzyme of cell wall and involved in modifying various physiological process for stress tolerance. Choa et al. have reported that constitutive expression of abiotic stress inducible hot pepper *CaXTH3*, which encodes Xyloglucanendo transglucosylase/hydrolase homolog and showed improved drought tolerance in transgenic *Arabidopsis* plants [84]. The same protein has been found to be increased in PDL-2 (tolerant) plant, through brassinosteroid induced plant hormone signal transduction pathway under drought stress in this study. Under drought conditions, plant needs to reduce shoot growth while maintaining root growth. This process requires differential cell wall synthesis and remodelling. Formation of reactive oxygen species and peroxidises are key players in this process, which initially cross link phenolic compounds and glycoproteins of the cell walls causing stiffening. Xyloglucan modifying enzymes results in cell wall loosening which allows further growth of stressed organs [85].

Absicisic acid dependent pathway can be considered important for drought tolerance in lentil as, gene encoding 9-cis-epoxycarotenoid dioxygenase which is a well known key enzyme for carotenoid biosynthesis, a precursor for ABA biosynthesis is highly upregulated in drought tolerant genotype under drought condition [86]. Increase in cellular ABA triggers the activation of several stress responsive genes and the closure of stomata to restrict transpiration [87, 88]. The same has been confirmed in several plant species including *Arabidopsis*, cowpea, beans, etc. [88–90]. Genes involved in lysine degradation were also upregulated in drought tolerant genotype. In plants, lysine is catabolised to glutamic acid and acetyl CoA with the help of two enzymes *viz.* lysine ketoglutarate reductase and saccharopine dehydrogenase, whose gene expression was also involved in response to ABA and drought stress in soybean [91]. WRKY33 is involved in drought stress regulation; this gene was found to be upregulated in tolerant genotype under drought condition. WRKY33 was reported to be directly associated with drought tolerance through transcriptional regulation of *Ces8A*, an *Arabidopsis* drought tolerant gene [92]. Drought tolerant genes like MYB, ZFP, Metallothiorenin and others identified in our findings were compared to the list of genes identified and were found matching to the Rice database as well [93].

## Conclusion

The present investigation report the transcriptome analysis of leaves and characterization of transcripts related to drought stress during the seedling stage in lentil using next generation sequencing approach. A total of 18,369 transcripts were expressed under drought stress and control conditions. These transcripts were successfully annotated by mapping them to KEGG pathway databases. qRT-PCR was used to validate the expression levels of 10 selected transcripts. The results show a close relationship between qRT-PCR and transcriptome data under drought stress conditions. Furthermore, SSRs (9949), SNPs (8260) and INDELs (1248) were identified successfully which can be further developed and serve as new resources for future genetic and functional genomics studies for drought tolerance in lentil. Above findings match with the phenotypic characterization of both the genotypes under drought stress, which exhibited higher relative water content, membrane stability index, proline, glycine betaine and enzyme activities and lower TBRAS contents in PDL-2 under drought stress compared to sensitive line JL-3. This is the first transcriptomic study on the response of lentil to drought stress at seedling stage.

## Additional files

Additional file 1: Figure S1. Number of upregulated and downregulated transcripts in different genotypes. (TIF 422 kb)
Additional file 2: Figure S2. Edward plot for Comparison of Contigs that are upregulated between all the samples. (TIF 2266 kb)
Additional file 3: Figure S3. Edward plot for Comparison of Contigs that are downregulated between all the samples. (TIF 2271 kb)
Additional file 4: Figure S4. BLASTX E-value **(**a**)** and BLASTX similarity score **(**b**)** distribution of transcriptome of all the combinations. (TIF 1125 kb)
Additional file 5: Table S1. Comparison of 1 T-1C Vs 2 T-2C with DEGs using Edge (R). (XLS 14709 kb)
Additional file 6: Figure S5. Number of Differentially Expressed Genes with Annotation plotted against Log2(Fold change) with *p* value < 0.05 in 1C_Control_1T_Treated (a), 2C Control_2 T_Treated (b), 2 T Treated_1T_Treated (c). (TIF 1021 kb)
Additional file 7: Figure S6. HeatMap of Top Up regulated DEGs between samples with *p* value < 0.05 in 1C_Control_1T_Treated. (TIF 1899 kb)
Additional file 8: Figure S7. HeatMap of Top Down regulated between samples with *p* value < 0.05 in 1C_Control_1T_Treated. (TIF 974 kb)
Additional file 9: Figure S8. HeatMap of Top Up regulated between samples with *p* value < 0.05 in 2C_Control_2T_Treated. (TIF 1532 kb)
Additional file 10: Figure S9. HeatMap of Top Down regulated between samples with *p* value < 0.05 in 2C_Control_2T_Treated. (TIF 872 kb)
Additional file 11: Figure S10. Number of GO Terms found in all the genotypes. (TIF 278 kb)
Additional file 12: Figure S11. Top 25 GO terms for down-regulated and up-regulated transcripts in ‘1 T’ as compared to ‘1C. (TIF 2218 kb)
Additional file 13: Figure S12. Top 25 GO terms for down-regulated and up-regulated transcripts in ‘2 T’ as compared to ‘2C’. (TIF 1756 kb)
Additional file 14: Table S2. Number of SNPs indentified using GATK toolkit Haplotype caller tool version 3.6-0 in different genotypes. (DOCX 11 kb)

## Abbreviations

ABA: Abscisic acid
APX: Ascorbate peroxidase
BLASTx: Basic Local Alignment Search Toolx
bp: Base pair
CANoPI: Contig Annotator Pipeline
CAT: Catalase
cDNA: Complementary DNA
ER: Endoplasmic reticulum
EST: Expressed sequence tags
FPKM: Fragments Per Kilobase of transcript per Million mapped reads
GO: Gene Ontology
GPX: Glutathione peroxidase
IARI: Indian Agricultural Research Institute
ICARDA: International Center for Agricultural Research in the Dry Areas
KEGG: Kyoto Encyclopedia of Genes and Genomes
KEGG: Kyoto Encyclopedia of Genes and Genomes
KOBAS: KEGG Orthology Based Annotation System
LEA: Late embryogenesis abundant
MDA: Malondialdehyde
MSI: Membrane stability index
NCBI: National Center for Biotechnology Information
NGS: Next Generation Sequencing
PCR: Polymerase chain Rreaction
qRT-PCR: Quantitative real-time PCR
RIN: RNA integrity number (RIN)
RNA: Ribonucleic acid
ROS: Reactive oxygen species
RWC: Relative water content
SNP: Single nucleotide polymorphisms
SOD: Superoxide dismutase
SSR: Simple sequence repeats
TF: Transcription factor
UniProt: Universal protein resource
