# Supplementary figures and images for: Transcriptome analysis of lentil (Lens culinaris Medikus) in response to seedling drought stress
Source: BMC Genomics. 2017 Feb 27;18:206. doi: 10.1186/s12864-017-3596-7 (PMC5327544; doi:10.1186/s12864-017-3596-7)

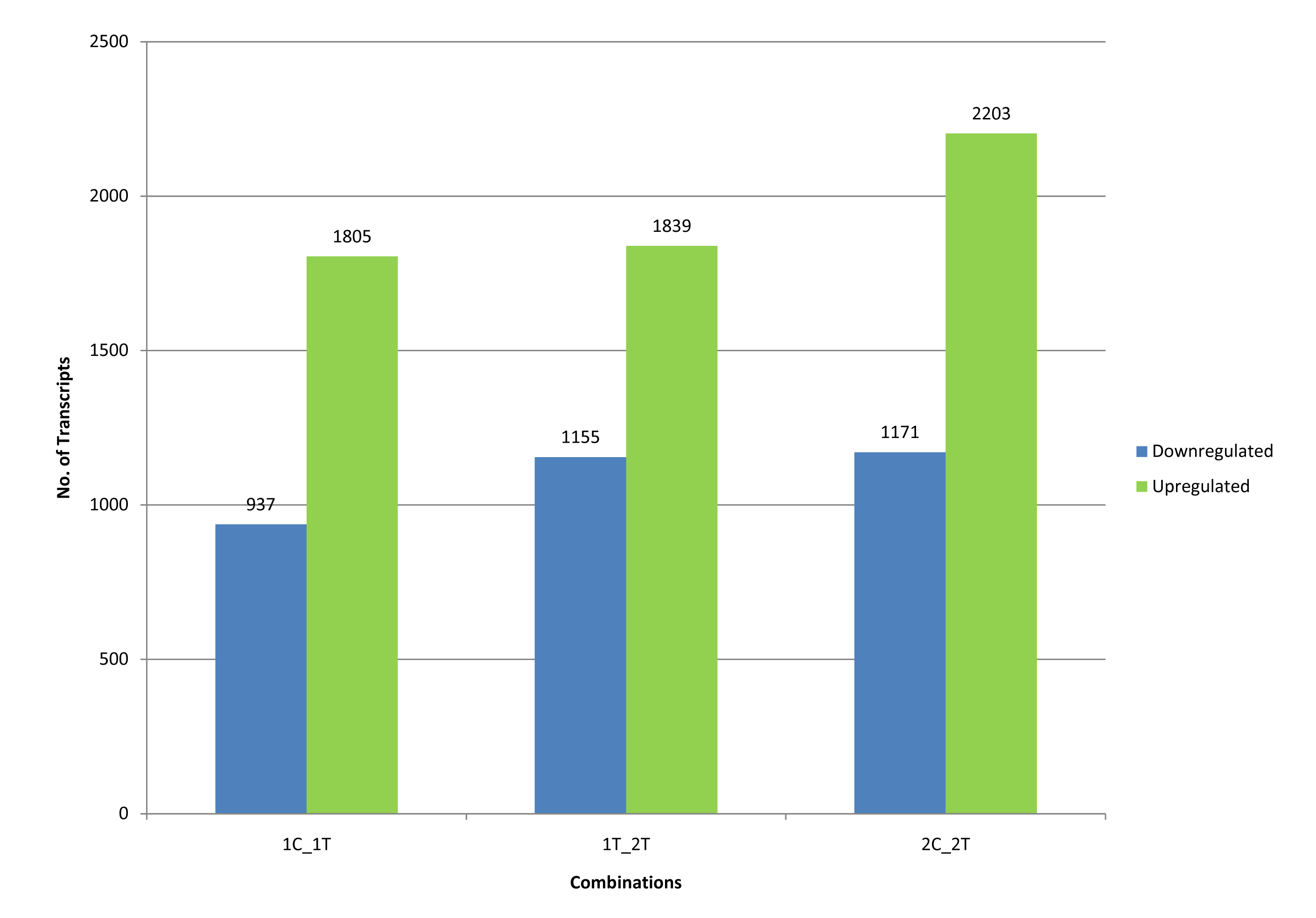

Supplement: Additional file 1: Figure S1. — Number of upregulated and downregulated transcripts in different genotypes. (TIF 422 kb) [file 12864_2017_3596_MOESM1_ESM.tif]

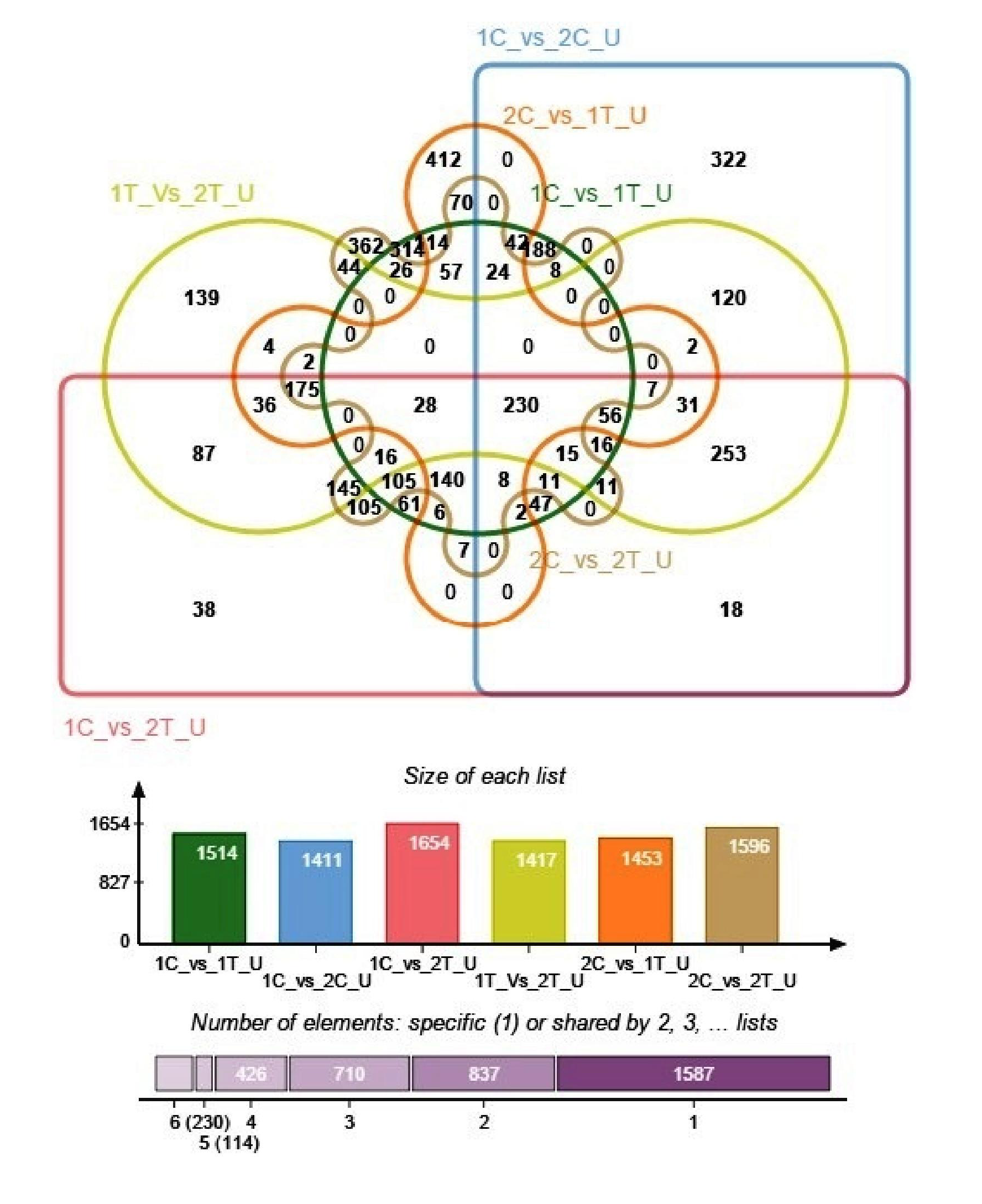

Supplement: Additional file 2: Figure S2. — Edward plot for Comparison of Contigs that are upregulated between all the samples. (TIF 2266 kb) [file 12864_2017_3596_MOESM2_ESM.tif]

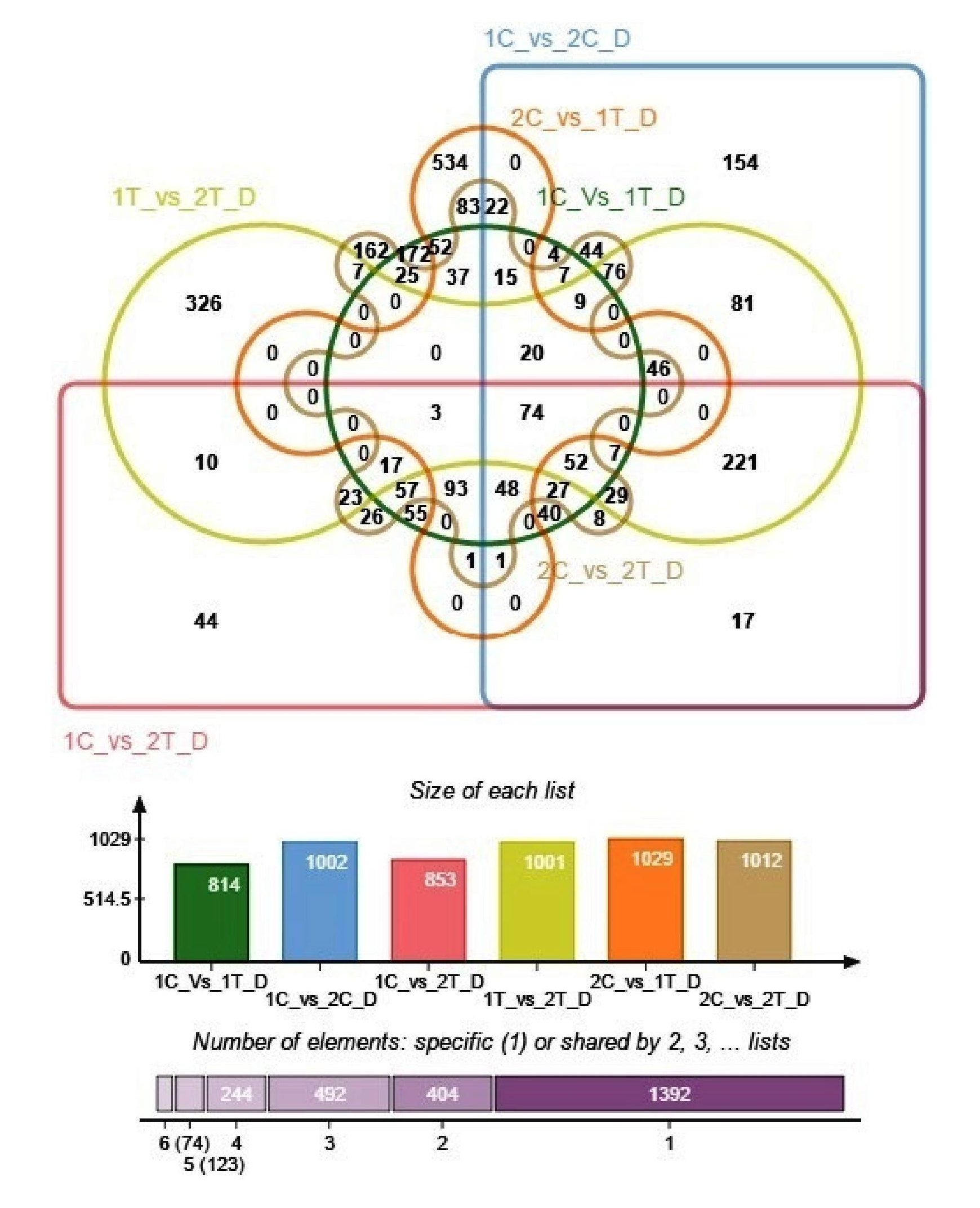

Supplement: Additional file 3: Figure S3. — Edward plot for Comparison of Contigs that are downregulated between all the samples. (TIF 2271 kb) [file 12864_2017_3596_MOESM3_ESM.tif]

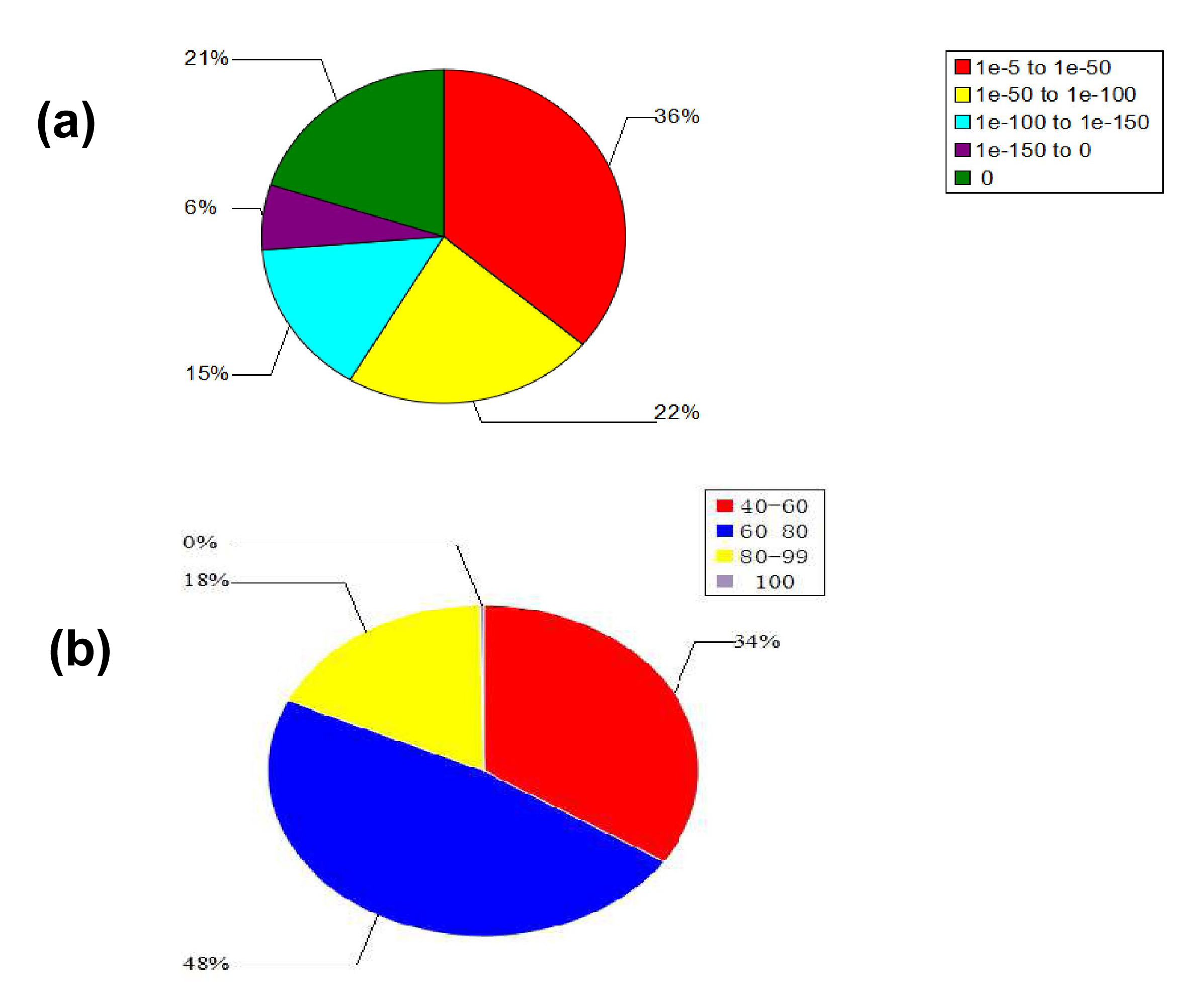

Supplement: Additional file 4: Figure S4. — BLASTX E-value (a) and BLASTX similarity score (b) distribution of transcriptome of all the combinations. (TIF 1125 kb) [file 12864_2017_3596_MOESM4_ESM.tif]

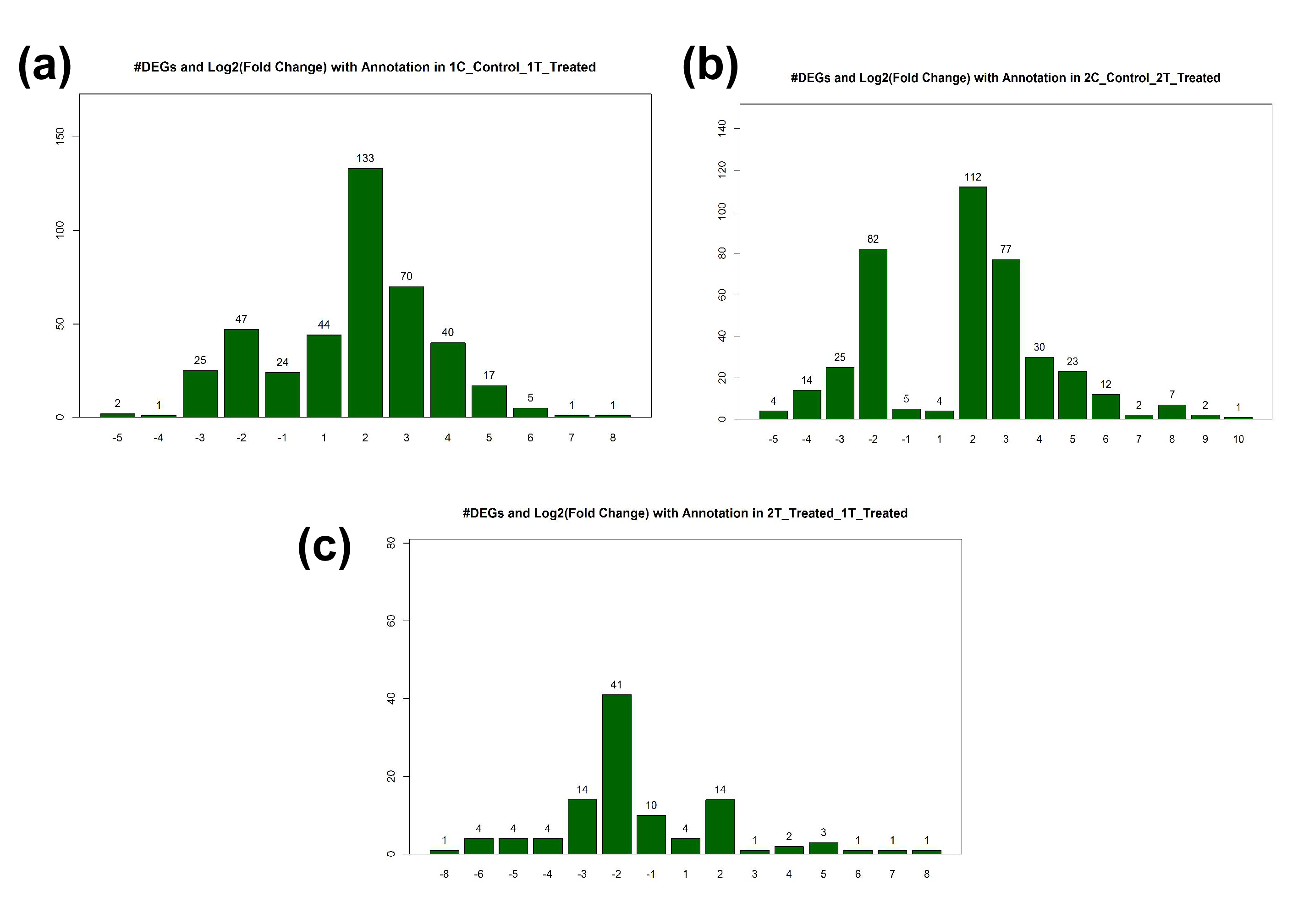

Supplement: Additional file 6: Figure S5. — Number of Differentially Expressed Genes with Annotation plotted against Log2(Fold change) with p value < 0.05 in 1C_Control_1T_Treated (a), 2C Control_2 T_Treated (b), 2 T Treated_1T_Treated (c). (TIF 1021 kb) [file 12864_2017_3596_MOESM6_ESM.tif]

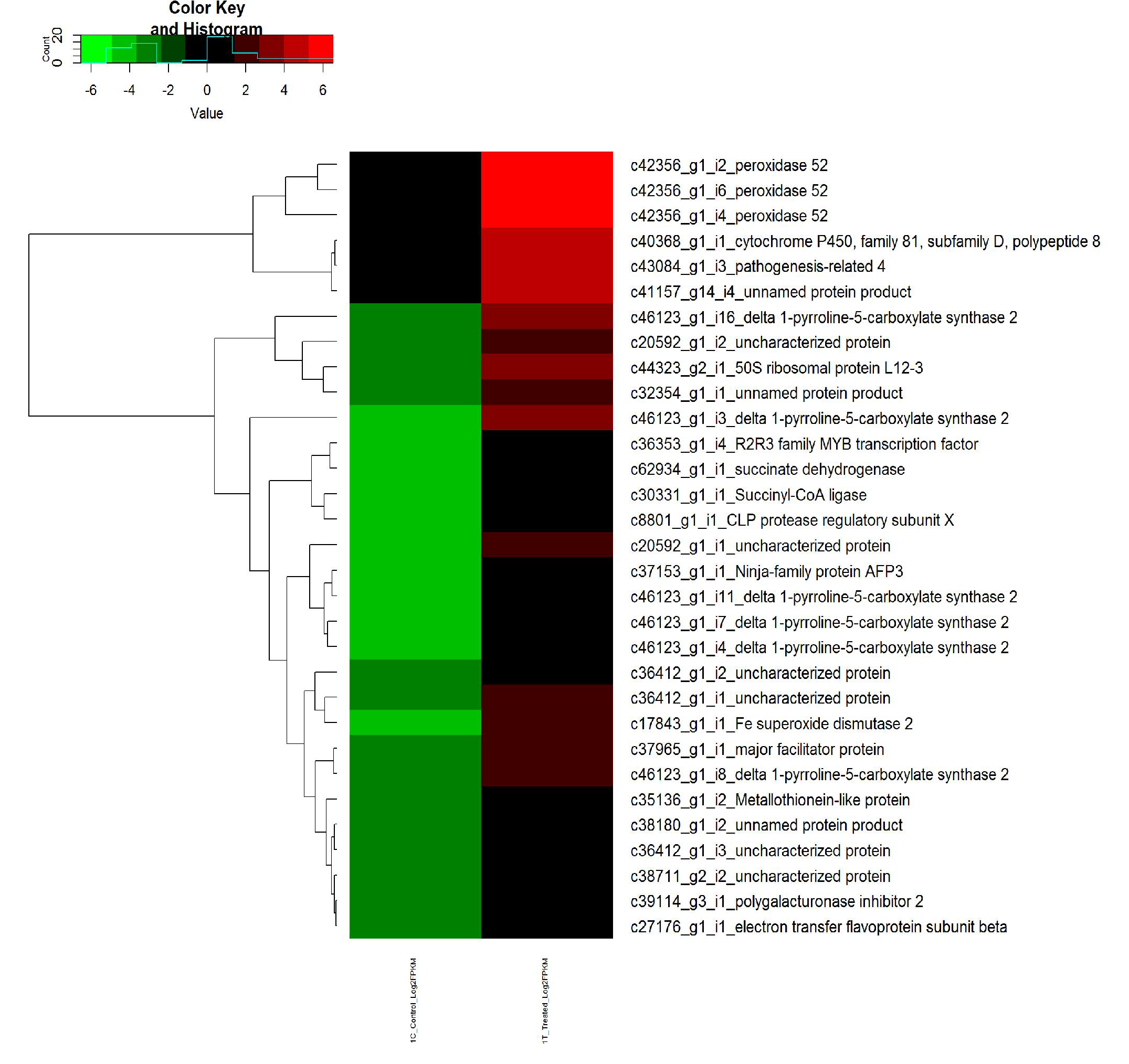

Supplement: Additional file 7: Figure S6. — HeatMap of Top Up regulated DEGs between samples with p value < 0.05 in 1C_Control_1T_Treated. (TIF 1899 kb) [file 12864_2017_3596_MOESM7_ESM.tif]

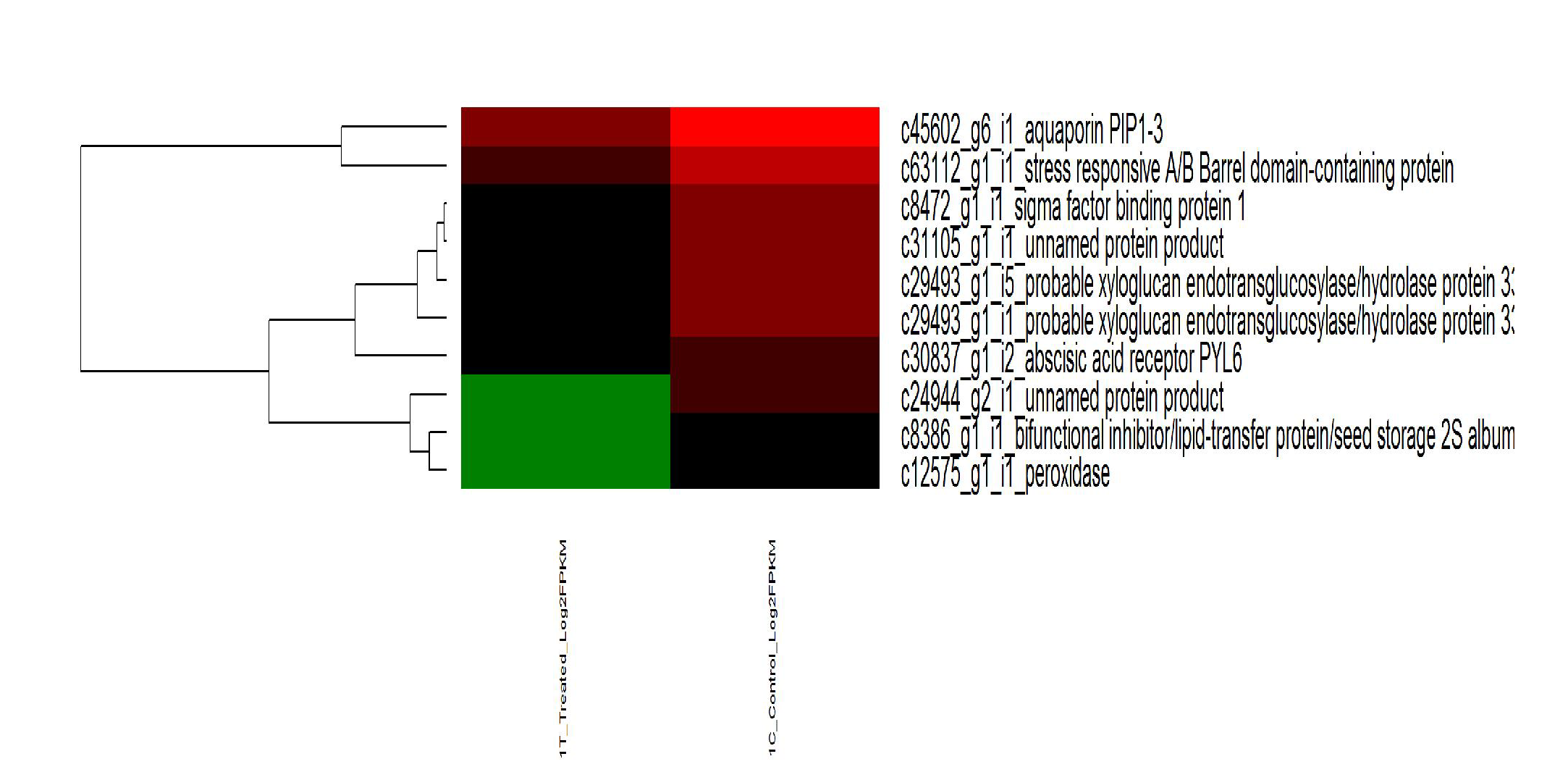

Supplement: Additional file 8: Figure S7. — HeatMap of Top Down regulated between samples with p value < 0.05 in 1C_Control_1T_Treated. (TIF 974 kb) [file 12864_2017_3596_MOESM8_ESM.tif]

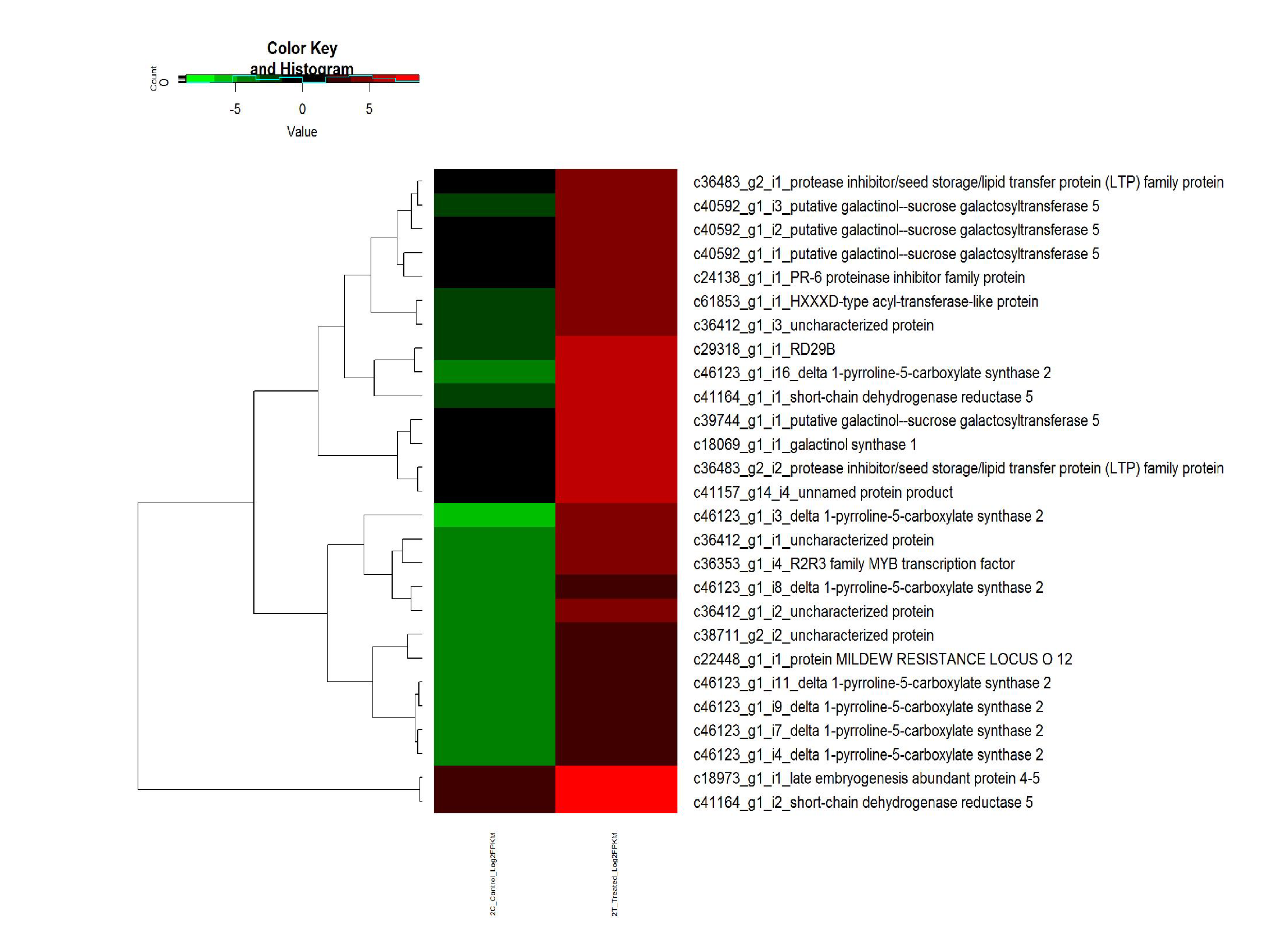

Supplement: Additional file 9: Figure S8. — HeatMap of Top Up regulated between samples with p value < 0.05 in 2C_Control_2T_Treated. (TIF 1532 kb) [file 12864_2017_3596_MOESM9_ESM.tif]

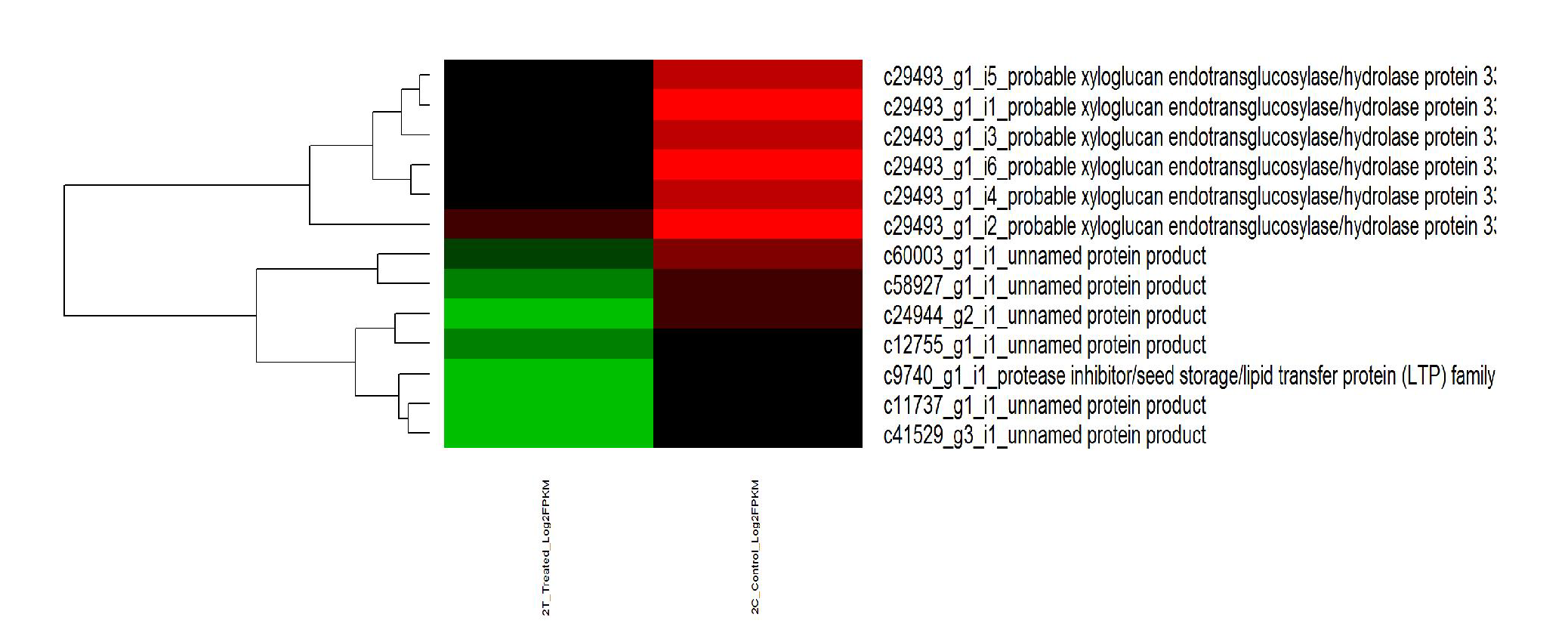

Supplement: Additional file 10: Figure S9. — HeatMap of Top Down regulated between samples with p value < 0.05 in 2C_Control_2T_Treated. (TIF 872 kb) [file 12864_2017_3596_MOESM10_ESM.tif]

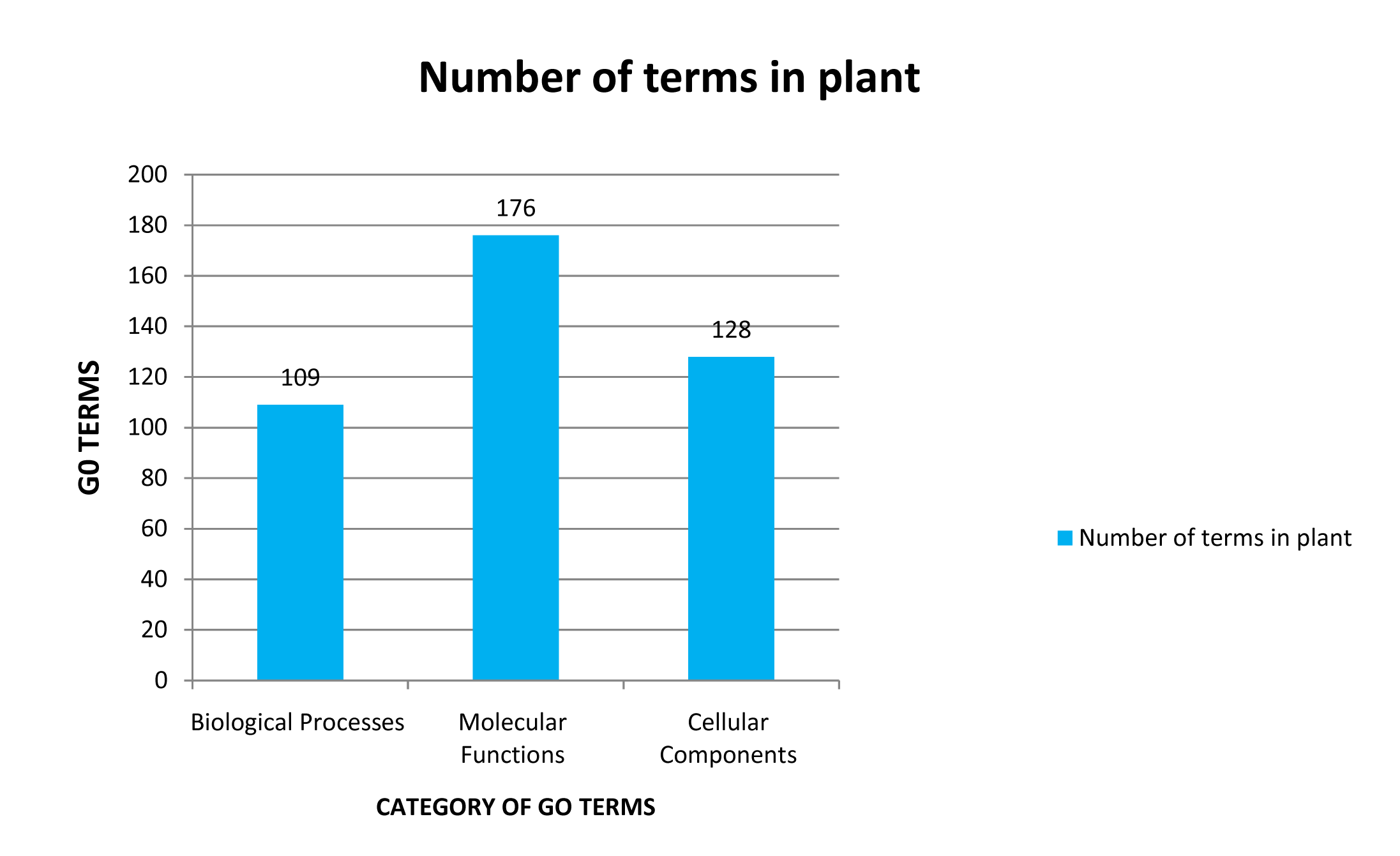

Supplement: Additional file 11: Figure S10. — Number of GO Terms found in all the genotypes. (TIF 278 kb) [file 12864_2017_3596_MOESM11_ESM.tif]

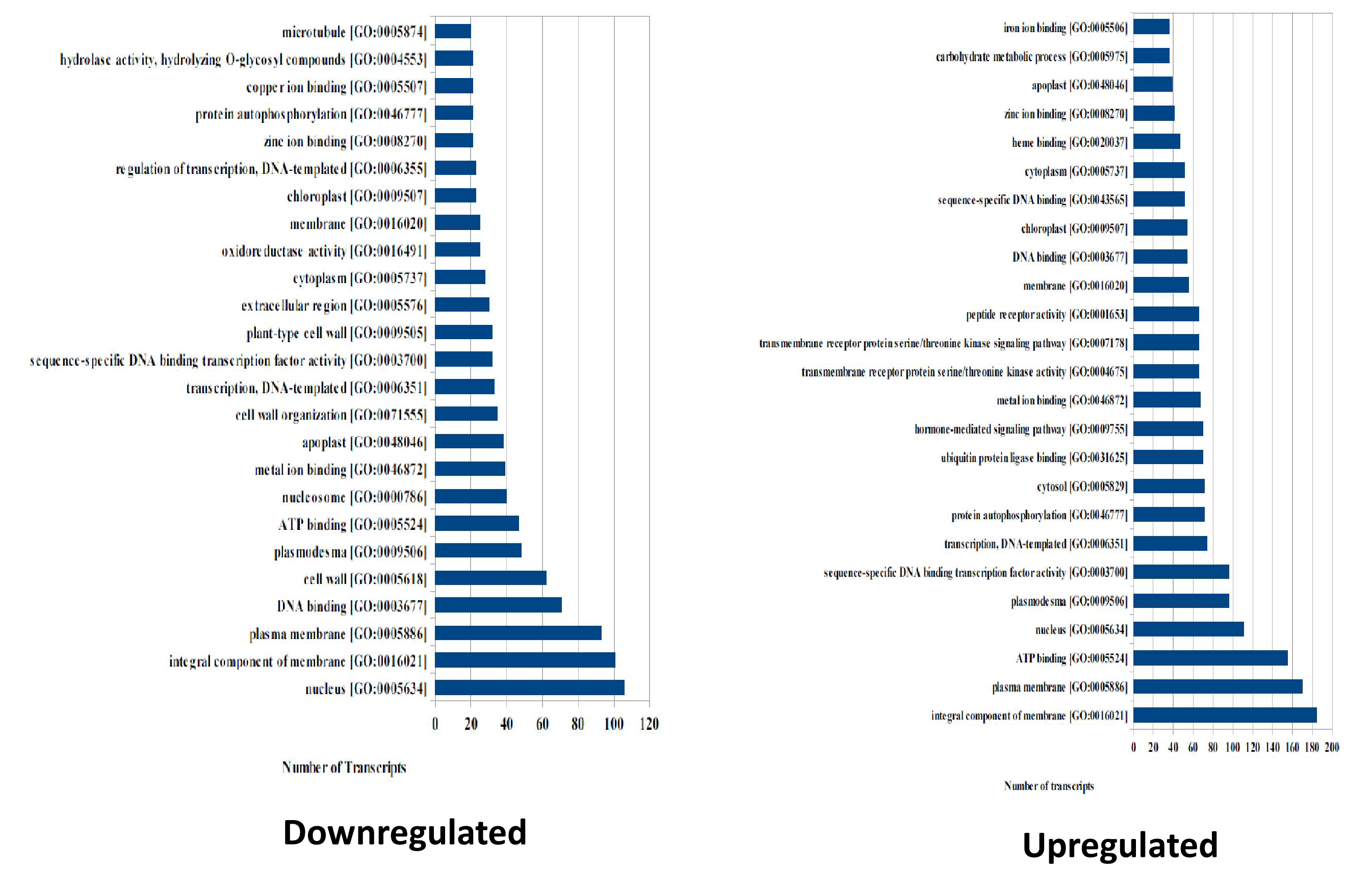

Supplement: Additional file 12: Figure S11. — Top 25 GO terms for down-regulated and up-regulated transcripts in ‘1 T’ as compared to ‘1C. (TIF 2218 kb) [file 12864_2017_3596_MOESM12_ESM.tif]

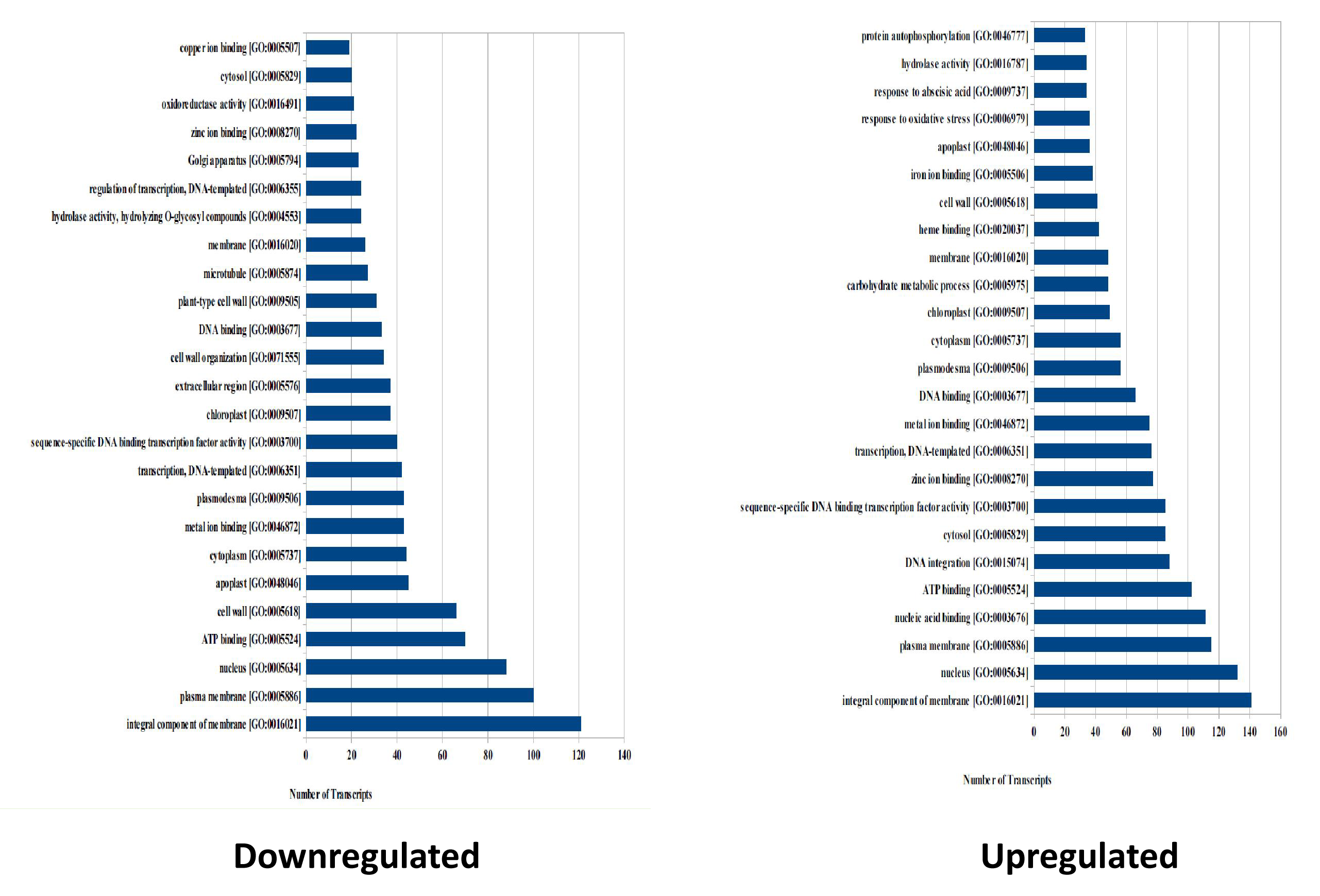

Supplement: Additional file 13: Figure S12. — Top 25 GO terms for down-regulated and up-regulated transcripts in ‘2 T’ as compared to ‘2C’. (TIF 1756 kb) [file 12864_2017_3596_MOESM13_ESM.tif]
