# Supplementary material for: Transcriptome analysis of lentil (Lens culinaris Medikus) in response to seedling drought stress
Source: BMC Genomics. 2017 Feb 27;18:206. doi: 10.1186/s12864-017-3596-7 (PMC5327544; doi:10.1186/s12864-017-3596-7)
Supplement: Additional file 14: Table S2. — Number of SNPs indentified using GATK toolkit Haplotype caller tool version 3.6-0 in different genotypes. (DOCX 11 kb) [file 12864_2017_3596_MOESM14_ESM.docx]

**Additonal file 14. Table 2.** Number of SNPs indentified using GATK toolkit Haplotype caller tool version 3.6-0 in different genotypes.

| **Sample** | **No. of SNPs** |
| --- | --- |
| 1C | 22900 |
| 1T | 24340 |
| 2C | 20432 |
| 2T | 28650 |
